# A theoretical approach on the ability of functionalized gold nanoparticles for detection of Cd^2+^

**DOI:** 10.1038/s41598-021-02933-5

**Published:** 2021-12-06

**Authors:** Mohammad Khavani, Aliyeh Mehranfar, Mohammad Izadyar

**Affiliations:** 1 Department of Chemistry and Materials Science, School of Chemical Engineering, Aalto University, P.O. Box 16100, 00076 Aalto, Finland; 2 Research Center for Modeling and Computational Sciences, Faculty of Science, Ferdowsi University of Mashhad, Mashhad, Iran

**Keywords:** Theoretical chemistry, Computational chemistry, Density functional theory, Molecular dynamics

## Abstract

Cadmium (Cd) as a toxic element that is widely present in water, soil, and air has important effects on human health, therefore proposing an accurate and selective method for detection of this element is of importance. In this article, by employing full atomistic molecular dynamics (MD) simulations and density functional theory dispersion corrected (DFT-D3) calculations, the effects of 6-mercaptonicotinic acid (MNA) and l-cysteine (CYS) on the stability of gold nanoparticles (AuNPs) and their sensitivity against Cd^2+^ were investigated. The obtained results indicate that pure AuNPs are not stable in water, while functionalized AuNPs with CYS and MNA groups have considerable stability without aggregation. In other words, the functional groups on the surface of AuNPs elevate their resistance against aggregation by an increase in the repulsive interactions between the gold nanoparticles. Moreover, functionalized AuNPs have considerable ability for selective detection of Cd^2+^ in the presence of different metal ions. Based on the MD simulation results, MNA-CYS-AuNPs (functionalized AuNPs with both functional groups) have the maximum sensitivity against Cd^2+^ in comparison with MNA-AuNPs and CYS-AuNPs due to the strong electrostatic interactions. DFT-D3 calculations reveal that the most probable interactions between the metal ions and functional groups are electrostatic, and Cd^2+^ can aggregate functionalized AuNPs due to strong electrostatic interactions with MNA and CYS groups. Moreover, charge transfer and donor–acceptor analyses show that molecular orbital interactions between the functional groups and Cd^2+^ can be considered as the driving force for AuNPs aggregation. A good agreement between the theoretical results and experimental data confirms the importance of the molecular modeling methods as a fast scientific protocol for designing new functionalized nanoparticles for application in different fields.

## Introduction

Today, due to rapid advancement in nanomaterial science and nanochemistry, a variety of nanosensors for selective detection of different compounds are available^1,2^. Currently, nanoscience provides many opportunities for designing nanosensors with high sensitivity based on nanomaterials^3,4^ including gold nanoparticles^5,6^, graphene^7^, carbon nanoparticles^8^, and nanopeptides^9^. Designing sensitive, specific, and inexpensive sensors for the detection of ions and compounds in different fields have been considered by scientists^10,11^.

Cadmium (Cd) as a toxic element is wildly present in air, soil, and water^12^. The targets of this element are lung, liver, kidney, bone, and immune system^13^. Cadmium is considered as a human carcinogen by the IARC (International Agency for Research on Cancer)^14^. Therefore, proposing an accurate method for selective detection of cadmium is of importance. Different methods have been proposed for the detection of Cd^2+^, such as electrochemical^15^ and spectroscopic methods^16–20^. These methods provide an acceptable sensitivity, but they are expensive, complicated, and time-consuming.

Rapid and accurate detection of toxic metal ions is of importance to combat environmental pollution. Functionalized gold nanoparticles have a key role in this by assisting the development of smart sensors and detection agents^21^. Colorimetric sensors based on functionalized gold nanoparticles (AuNPs) can be good candidates for the detection of Cd^2+^ with considerable sensitivity and selectivity. There are many theoretical and experimental reports in the literature about the application of the functionalized AuNPs for selective detection of different compounds^22–27^. For example, the designed sensor based on triazole functionalized gold nanoparticles by Wang and coworkers shows a considerable sensitivity against dopamine^28^. The obtained results indicate that this sensor can detect dopamine in the presence of histidine, tyrosine, epinephrine, isopernaline, norepinephrine, and serotonin with a 0.07 µM limit of detection (LOD) value. Wei et al. reported functionalized AuNPs by carboxymethyl cellulose for selective detection of cysteine as a colorimetric sensor^29^. Based on the obtained results, cysteine causes the aggregation of the functionalized AuNPs due to considerable interactions with carboxymethyl cellulose groups, while other amino acids, such as alanine, histidine, phenylalanine, and arginine cannot aggregate the nanoparticles. Moreover, Sener and coworkers designed a colorimetric sensor for the detection of heavy metal ions (Hg^2+^, Fe^3+^, Pb^2+^, Ca^2+^, and Cr^3+^) in water^30^. The designed sensor by this group is composed of different functional groups. The functional groups on the surface of AuNPs have a key role on the selectivity of the proposed sensor. For example, functionalized AuNPs with 11-mercaptoundecanoic acid (MUA) and lysine amino acid are selective toward Pb^2+^, while nanoparticles with MUA and histidine groups can detect Hg^2+^ in the presence of other ions. The shape of the gold nanoparticles is another factor that can be important in the sensitivity of the colorimetric sensors. In this context, the reported sensor based on the functionalized gold nanorods by Politi et al. shows more sensitivity against Pb^2+^ in comparison with other spherical functionalized gold nanoparticles^30,31^. In addition to the experimental researches, theoretical studies have been performed on the molecular properties of functionalized AuNPs^32,33^. Tavanti et al. by employing theoretical methods investigated the interactions between the functionalized AuNPs and amyloid-β fibrils^34^. Based on the results, this nanoparticle interacts with amyloid-β fibril through the hydrophobic interactions and monolayer protected nanoparticle is a good candidate to prevent fibril aggregation. It is possible to combine functionalized gold nanoparticles with other nanomaterials to design electrochemical sensors. In this contexts, Cheng et al. designed an electrochemical sensor based on the functionalized AuNPs with cysteine and nitrogen-doped graphene for detection of Pb^2+^^35^. Their obtained results confirmed that functionalized gold nanoparticles increase the sensitivity of the electrochemical sensor in comparison with other modified electrodes and these nanoparticles have a key on the selectivity of the designed electrode against Pb^2+^.

In addition to the gold nanoparticles, functionalized silver nanoparticles have also a considerable ability for selective detection of different metal ions. For example, Kailasa et al. designed sensors based on functionalized silver nanoparticles with 6-mercaptonicotinic acid and melamine as a colorimetric sensor for Cr^3+^ and Ba^2+^ ions^36^. The color of the solution changes from yellow to reddish-brown and orange in the presence of Cr^3+^ and Ba^2+^, respectively, due to the aggregation of the functionalized nanoparticles. Based on the obtained results, these modified silver nanoparticles acted as a colorimetric sensor for Cr^3+^ and Ba^2+^ ions, providing a limit of detection of 64.51 nM and 80.21 nM for Cr^3+^ and Ba^2+^ ions, respectively. Moreover, the reported colorimetric sensor based on the functionalized silver nanoparticles with cysteine groups by Venditti et al. shows a considerable sensitivity and selectively against Hg^2+^^37^. Their obtained results confirmed that this sensor has 1 ppm limit of detection and can detect Hg^2+^ in the presence of different metal ions, selectively.

In this article to have a molecular insight into the effect of functional groups on the sensing properties of AuNPs, dynamical behavior and sensing ability of the functionalized AuNPs by 6-mercaptonicotinic acid (MNA) and _L_-cysteine (CYS) against Cd^2+^ were investigated by employing molecular dynamics (MD) simulations and density functional theory dispersion corrected (DFT-D3) calculations. Cadmium as a toxic element has devastating effects on human health, therefore detecting this element is of importance. In this context, Xue et al. reported functionalized AuNPs by 6-mercaptonicotinic acid (MNA) and _L_-cysteine (CYS) groups for selective detection of Cd^2+^ in the presence of Ba^2+^, Ca^2+^, Mg^2+^, and Pb^2+^^38^. Since the mechanism of Cd^2+^ detection by these nanoparticles is unknown, theoretical studies on the interactions of Cd^2+^ and functionalized AuNPs can provide interesting details from the molecular viewpoint, which can explain the experimental observations. Moreover, the importance of this study is that by using molecular modeling methods, it is possible to design nanoparticles with different functional groups for selective detection of different chemicals.

## Computational details

### Molecular dynamics simulations

To investigate the effects of MNA and CYS on the sensing properties and dynamical behavior of gold nanoparticles (AuNPs), full atomistic molecular dynamics (MD) simulations were applied. The diameter of the corresponding nanoparticle is 2 nm. The surface of the AuNPs was covered completely by thirty functional groups. The functionalized AuNPs with (MNA)_30_, (CYS)_30_, and (MNA)_15_(CYS)_15_ are denoted as MNA-AuNP, CYS-AuNP, and MNA-CYS-AuNP, respectively. At the first step, six AuNPs were distributed in a cubic box of water (TIP3P water model^39^) with a minimum distance of 15 Å from each other, randomly. According to experimental conditions^38^ the functionalized AuNPs have greater sensitivity at pH = 10, therefore the deprotonated states of functional groups (MNA and CYS) were considered in the MD simulation section (Fig. S1).

To investigate the effect of CYS and MNA groups on the stability of the corresponding AuNPs, the dynamical behavior of six functionalized AuNPs was analyzed. To study the sensing ability of MNA-AuNPs, CYS-AuNPs and MNA-CYS-AuNPs, 200 ions of Ba^2+^, Cd^2+^, Ca^2+^, Mg^2+^, and Pb^2+^ were added around the functionalized AuNPs, randomly, with a minimum distance of 4 Å. To neutralize the system, Cl^−^ and Na^+^ ions were added. To exclude the initial conditions in the sensing ability and aggregation process of the functionalized gold nanoparticles, the initial positions of the pure and functionalized AuNPs were equally considered in all simulations. At the first step of MD simulation, energy minimization was performed on each system during 100,000 steps. Then, the temperature of the system increased from 0 to 300 K during 5000 ps (with 1 fs time step) MD simulations by applying an NVT ensemble, in which a restraining force constant of 2.5 kcal mol^−1^ was applied for solute structure. After this step, all systems were equilibrated during 10 ns NPT (1 bar and 300 K) MD simulations with 1 fs time step without any restraining force. Finally, 50 ns MD simulations in an NPT ensemble were performed with 2 fs time step on all obtained structures from the equilibration step as the product step.

All MD simulations were performed using Amber 14.0 software package^40^. General Amber Force Field (GAFF)^41^ parameters were used for MNA and CYS groups and reported parameters by Li et al.^42^ were employed for metal ions. Force field parameters, reported by Heinz et al.^43^, were applied for gold nanoparticle simulations and Au–S bond (S atom of the MNA and CYS groups) parameters were obtained from the Groenhof et al. report^44^. Geometry optimization at the M06-2X-D3/6-311++G(d,p) level of the theory was applied to obtain the geometrical parameters of MNA and CYS^45,46^. Atomic charges were calculated by the CHelpG method at the same level. The pressure was regulated by the isotropic Berendsen method (with a relaxation time of 1 ps) and the temperature was controlled using Langevin thermostat with a collision frequency of 1 ps^−1^^47,48^. To calculate the long range electrostatic interactions, Particle Mesh Ewald (PME) with 10 Å direct cutoff was employed^49^. SHAKE constraints were used on all bonds involving hydrogen atoms^50^.

### Quantum mechanics calculations

DFT-D3 calculations were applied to study the interactions between MNA and CYS with different metal ions. The structures of MNA- and CYS-metal ion complexes were optimized by the M06-2X-D3 functional^45^ and 6–311++G(d,p) basis set^46,51^ for all non-metallic elements, Cu^2+^, Ca^2+^ and Mg^2+^ ions. The effective core potential basis set of LANL2DZ was applied for Ba^2+^, Cd^2+^, Pb^2+^, Hg^2+^ and Au^52^. Frequency calculations were applied to calculate the thermodynamic parameters of MNA- and CYS-metal ion complexes. The effect of water on the corresponding complexes was considered by employing the conductor-like polarizable continuum model (CPCM)^53^.

To calculate the electrostatic interactions and charge transfer between the receptor groups and metal ions, natural bond orbital (NBO) analysis was applied^54^. All quantum mechanics (QM) calculations were performed using the Gaussian 09 computational package^55^. To compare the strength of interactions between the receptor groups and metal ions, localized orbital locator (LOL)^56^, electron localization function (ELF)^57^ 2D and 3D of non-covalent interaction (NCI) analyses^58^ were performed using the MultiWFN 3.7^59^.

## Results and discussion

### Effect of functional groups on the stability of AuNPs

To investigate the effect of CYS and MNA on the stability and dynamical behavior of gold nanoparticles, full atomistic MD simulations were applied. On the basis of the experimental results^38^, pure AuNPs are not stable in water, while the functionalized AuNPs show remarkable stability in the water against aggregation. Therefore study the effect of functional groups on the stability and dynamical behavior of the AuNPs is of importance.

**Figure 1:**
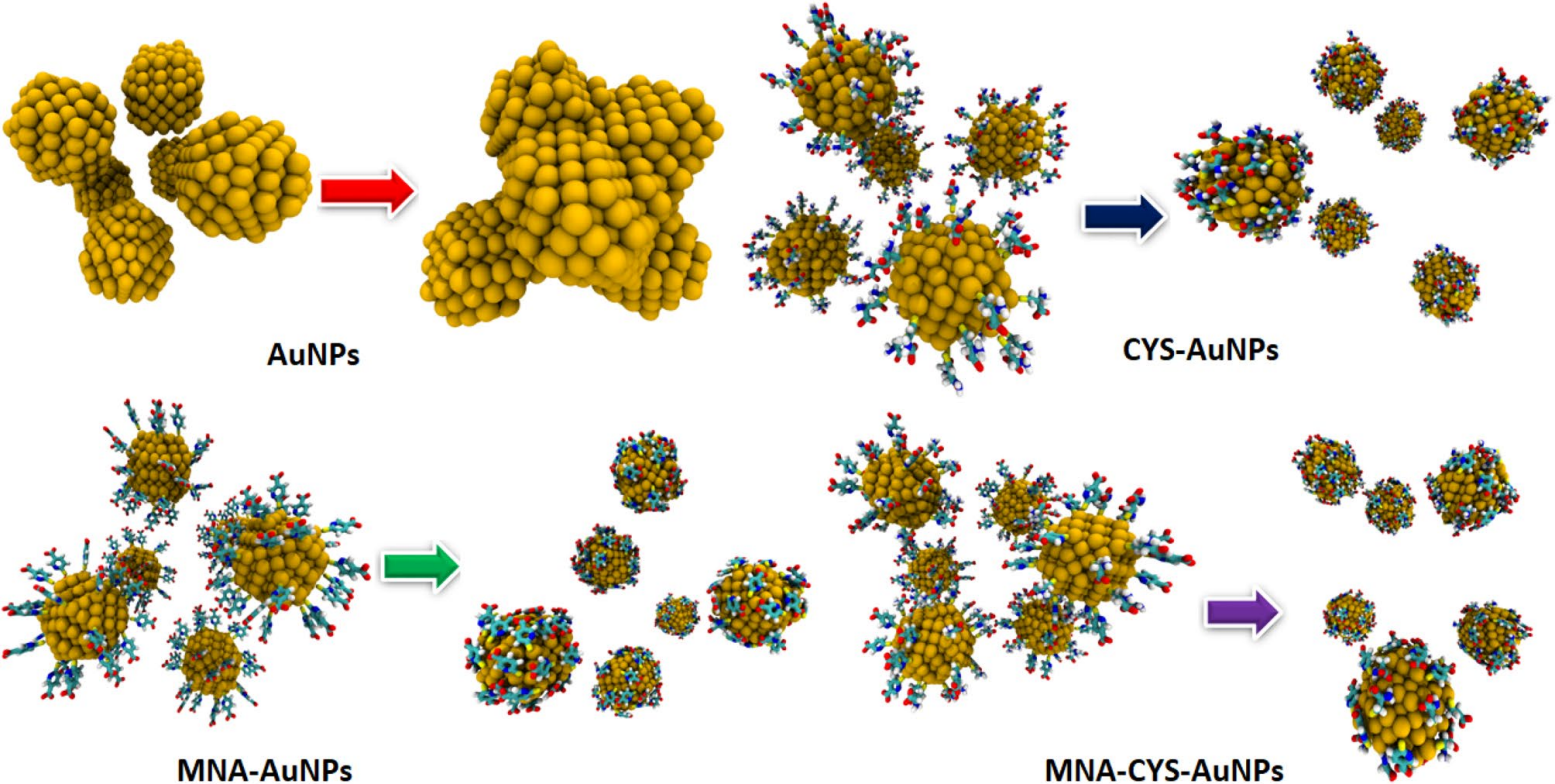
The initial and obtained structures of the pure and functionalized AuNPs after simulation time in water.

**Figure 2:**
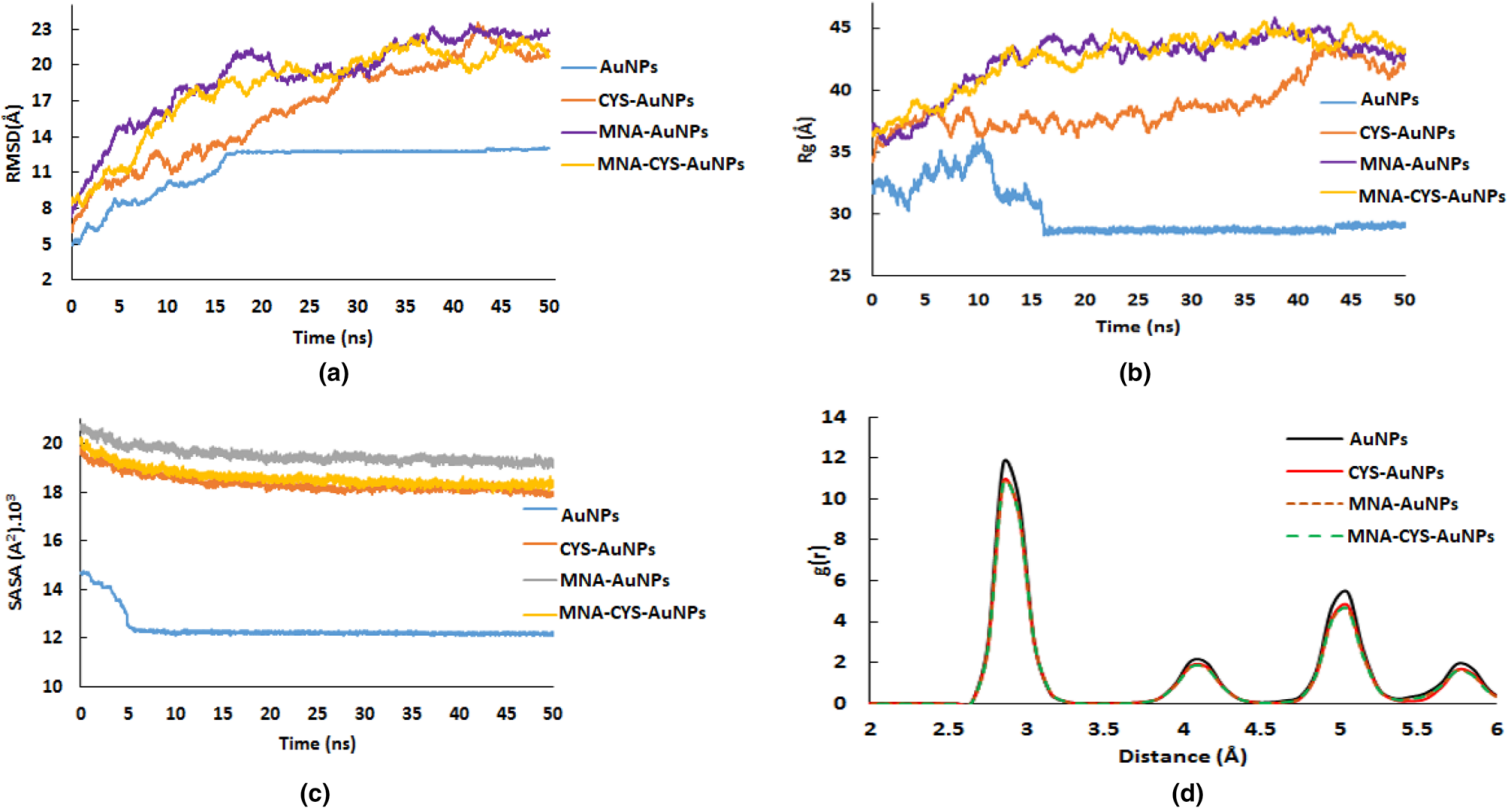
The calculated RMSD (**a**), Rg (**b**), and SASA (**c**) of the pure and functionalized AuNPs during the simulation time and RDF plot (**d**) of the Au–Au pair of the corresponding nanoparticles.

Figure 1 shows the obtained structures of AuNPs, CYS-AuNPs, MNA-AuNPs and MNA-CYS-AuNPs after the simulation time in water. According to this figure, pure AuNPs are fully aggregated, while functionalized AuNPs are stable according to the experimental results^38^. In other words, the obtained stable structures of the functionalized AuNPs in water confirmed the main role of the functional groups on the stability and dynamical behavior of AuNPs. According to the calculated root mean square deviation (RMSD) values in Fig. 2-a, pure AuNPs are fully aggregated after 15 ns MD simulations, while the calculated RMSD values of the functionalized nanostructures show an incremental trend with increasing the simulation time. Moreover, the calculated average values of RMSD confirm that MNA-AuNPs are more stable in comparison with CYS-AuNPs and MNA-CYS-AuNPs. This result indicates that the chemical structure of the functional group has an important effect on the stability and dynamical behavior of AuNPs. Therefore it is possible to change or tune up the stability and properties of the AuNPs by a change in the functional groups.

To analyze the role of MNA and CYS groups on the effective size of the gold nanoparticles, the radius of gyration (Rg) values were calculated during the simulation time (Fig. 2-b). According to Fig. 2-b, pure gold nanoparticles have a smaller size in comparison with the functionalized AuNPs. In other words, due to aggregation, the effective size of the pure nanoparticles reduces. On the other hand, the functional groups increase the effective size of the gold nanoparticles by increasing the resistance of AuNPs against aggregation, which is according to the RMSD and structural analyses. The aggregation process reduces the surface of the gold nanoparticles. A reduction in the surface of AuNPs decreases the energy of the system, which can be considered as the driving force for AuNPs aggregation.

The calculated solvent accessible surface area (SASA) of the pure and functionalized AuNPs confirms the aggregation of the AuNPs in water (Fig. 2-c). The calculated average SASA values of the pure AuNPs, CYS-AuNPs, MNA-AuNPs and MNA-CYS-AuNPs are 12.45 ± 0.56 × 10^3^, 18.37 ± 0.36 × 10^3^, 19.53 ± 0.32 × 10^3^ and 18.62 ± 0.38 × 10^3^ A^2^, respectively. The values of the SASA reveal that MNA-AuNPs have greater stability in water than other functionalized AuNPs. Based on the SASA values, functionalized AuNPs have greater solubility in water than pure AuNPs.

MNA and CYS groups on the gold nanoparticle surface increase the solubility of these AuNPs due to the hydrogen bond (H-bond) interactions with water molecules. The calculated average number of the H-bonds with water molecules for a single CYS-AuNPs, MNA-AuNPs and MNA-CYS-AuNPs during the simulation time are 222 ± 22, 291 ± 29 and 259 ± 26, respectively. On the basis of this result, MNA-AuNPs form the highest number of H-bond interactions with water molecules in comparison with other functionalized AuNPs. RMSD and Rg analyses confirmed that MNA-AuNPs have greater stability in water than CYS-AuNPs and MNA-CYS-AuNPs. This stability may be related to the greater H-bond formation between this nanoparticle and water molecules. In addition to the H-bond interactions, MNA and CYS groups on the AuNPs surface have a negative charge (COO^−^ group) that increases the electrostatic repulsion between the AuNPs and elevates their stability against aggregation.

The optical properties of gold nanoparticles depend on the shape, size, and metalcore characters. To analyze the effect of the functional groups on the properties of AuNPs, radial distribution function (RDF) analysis was applied. Figure 2-d shows the calculated RDFs for the Au–Au pair of the pure and functionalized AuNPs. This figure shows a first sharp peak at 2.8 Å for the Au–Au pair of the corresponding nanoparticles. On the other hand, the shapes of these RDFs for all nanoparticles (pure and functionalized) are really equal, which confirms that functional groups can only change the surface properties of the gold nanoparticles.

Overall, the van der Waals (vdW) interactions between the pure AuNPs increase the aggregation. On the other hand, negatively charged groups (MNA and CYS) elevate the electrostatic repulsion between the AuNPs, which increases their resistance against aggregation. In other words, the functionalization process of the gold nanoparticles by MNA and CYS groups increases their stability through the repulsive interaction mechanism.

### Cd^2+^ detection by functionalized AuNPs

The carboxylate group of MNA and CYS on the surface of AuNPs is the center of complex formation with metal ions. This group makes the functionalized gold nanoparticles a good candidate for metal ion detection. According to the experimental results^38^, MNA-AuNPs, CYS-AuNPs, and MNA-CYS-AuNPs can detect Cd^2+^ in the presence of different metal ions. To investigate the detection ability of these functionalized AuNPs against Cd^2+^ from the molecular viewpoint, their dynamical behavior was studied in the presence of different metal ions such as Ba^2+^, Ca^2+^, Mg^2+^, and Pb^2+^. It is well worth mentioning that experimental data about the sensing ability of the functionalized AuNPs are available for seventeen metal ions^38^. Since the analysis of dynamic behavior of the functionalized AuNPs in the presence of seventeen metal ions is theoretically very time-consuming, only the mentioned metal ions were selected as the possible candidates.

**Figure 3:**
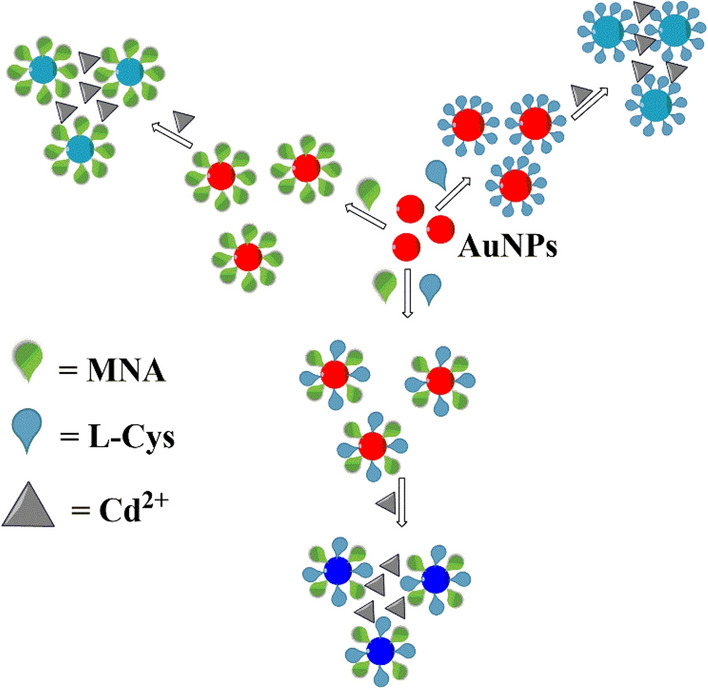
The proposed mechanism of Cd^2+^ detection by different functionalized AuNPs.

Figure 3 shows the mechanism of Cd^2+^ detection by the functionalized AuNPs with different functional groups. According to this figure, Cd^2+^ can eliminate the repulsive interactions of the functionalized AuNPs and induces them to aggregate due to strong interactions with CYS or MNA groups on the surface of AuNPs. The aggregation process changes the solution color from red to dark blue.

**Figure 4:**
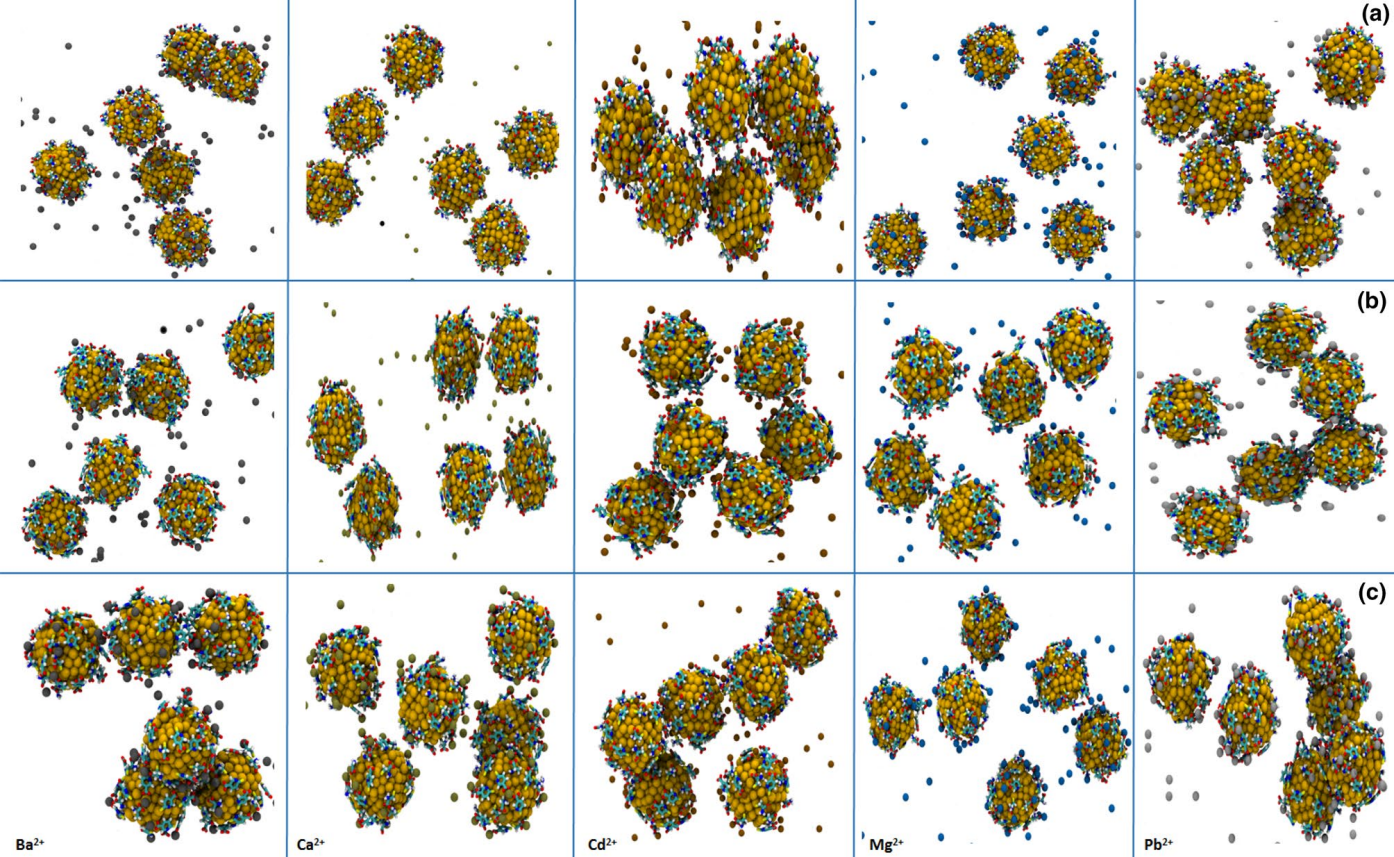
The obtained structures of CYS-AuNPs (**a**), MNA-AuNPs (**b**), and MNA-CYS-AuNPs (**c**) after 50 ns MD simulations in the presence of different metal ions.

Figure 4 illustrates the obtained structures of the functionalized AuNPs after 50 ns MD simulations in the presence of different metal ions. According to this figure, MNA-AuNPs, CYS-AuNPs, and MNA-CYS-AuNPs have the maximum aggregation in the presence of Cd^2+^ in comparison with other metal ions. Moreover, these functionalized AuNPs show a considerable amount of aggregation against Pb^2+^, which is according to the experimental results^38^. Furthermore, based on the experimental data, Mg^2+^ cannot aggregate the functionalized AuNPs that the obtained structures after simulations are in good agreement with the experimental observations.

**Figure 5:**
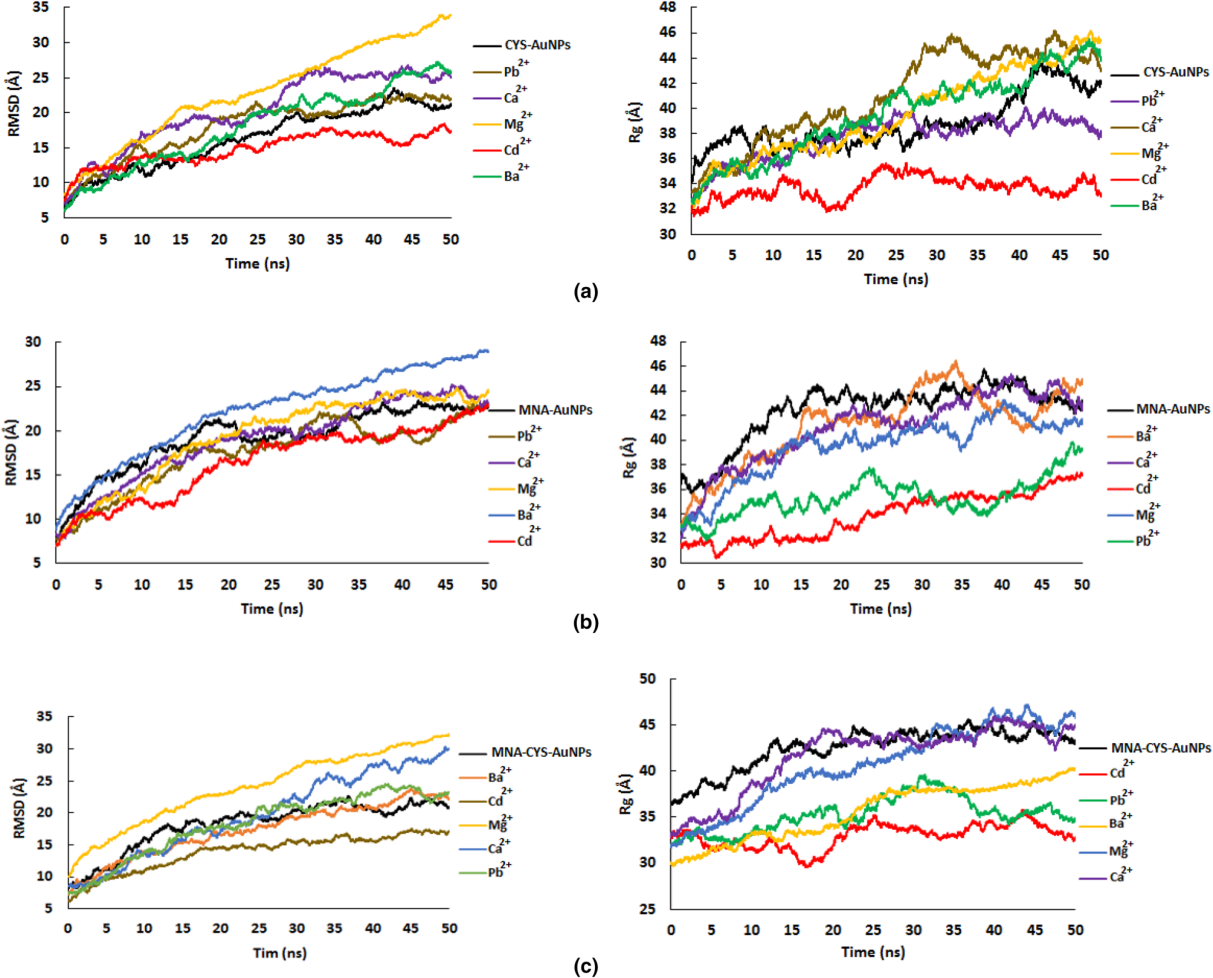
The calculated RMSD and Rg values of CYS-AuNPs (**a**), MNA-AuNPs (**b**), and MNA-CYS-AuNPs (**c**) in the presence of different metal ions.

To study the aggregation process of the functionalized AuNPs, RMSD, and Rg analyses were applied. According to Fig. 5, all functionalized AuNPs have the minimum RMSD value in the presence of Cd^2+^ confirming the aggregation process. Moreover, on the basis of the calculated RMSD values, CYS-AuNPs and MNA-CYS-AuNPs have the lowest interaction with Mg^2+^, while MNA-AuNPs show this behavior in the presence of Ba^2+^. The calculated Rg values (Fig. 5) reveal that functionalized AuNPs have the maximum of compactness in the presence of Cd^2+^ in comparison with other metal ions confirming their full aggregation.

**Figure 6:**
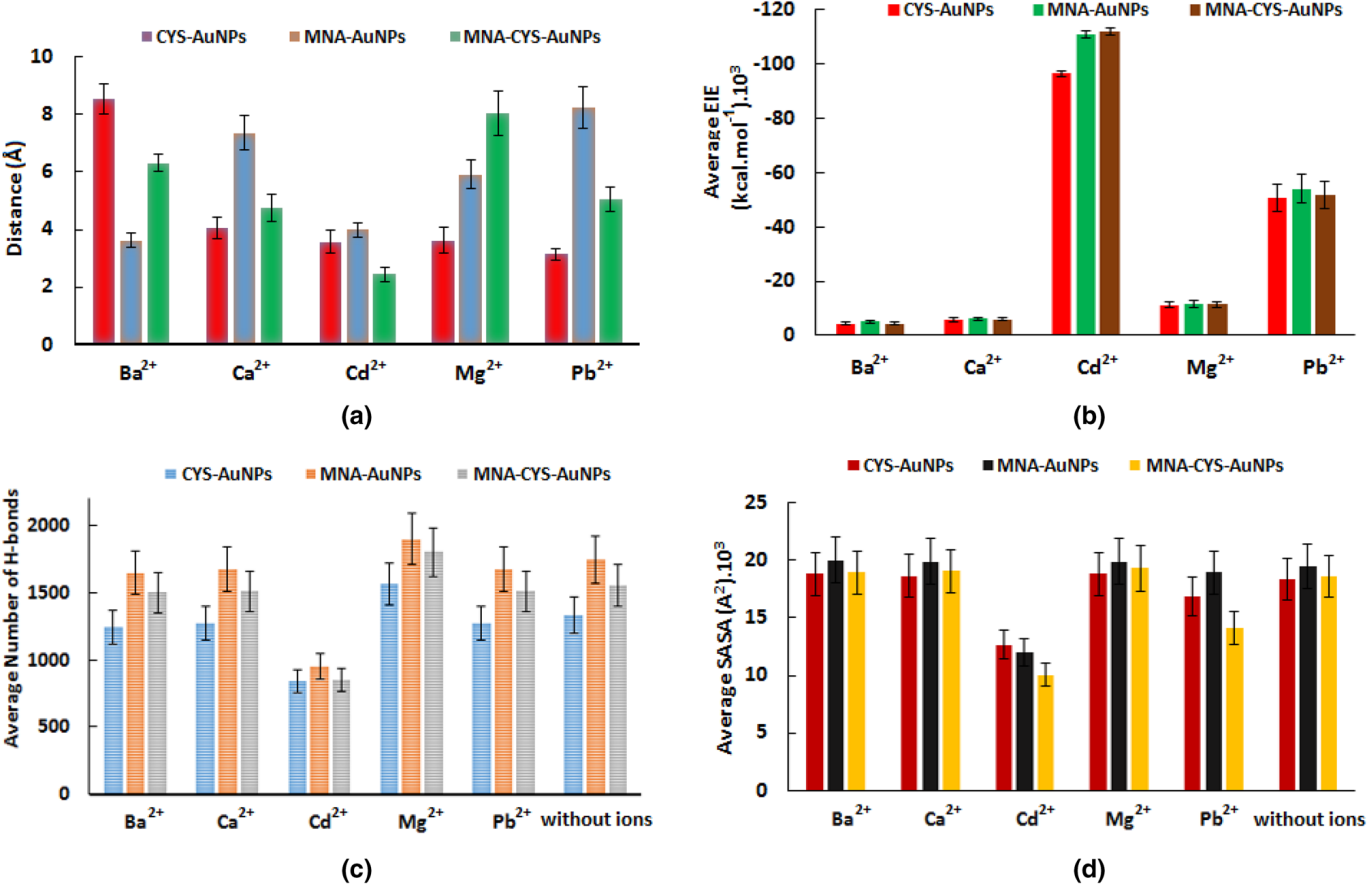
The calculated average distance (**a**) and average EIE (**b**) between the O atoms (of COO^−^ group of MNA and CYS) and different metal ions and the average H-bond (**c**) between the functional groups and water molecules and the calculated SASA of the functionalized AuNPs (**d**) in the presence and absence of different metal ions. The average values were calculated during 50 ns MD simulations and the error bars show the calculated standard deviation of the corresponding quantities.

Figure 6-a shows the calculated average distance between different metal ions and the COO^−^ group of MNA and CYS on the surface of functionalized AuNPs. Based on the calculated values, all functionalized AuNPs have the maximum interaction with Cd^2+^ in comparison with other metal ions. Experimental data reveal that MNA-CYS-AuNPs have higher sensitivity against Cd^2+^ in comparison with MNA-AuNPs and CYS-AuNPs^38^. According to the calculated average values of distance, MNA-CYS-AuNPs have the minimum distance or maximum of interaction with Cd^2+^ among the studied functionalized AuNPs, which confirms higher sensitivity for this nanoparticle according to experimental results^38^.

The calculated average values of electrostatic interaction energy (EIE) for the O atoms of the COO^−^ group of MNA and CYS with metal ions indicate that the functionalized AuNPs have stronger electrostatic interactions with Cd^2+^ than other metal ions (Fig. 6-b). Due to strong electrostatic interactions with functionalized AuNPs, Cd^2+^ can eliminate the repulsive interactions of these nanoparticles, which leads to aggregation. Other ions cannot overcome these interactions due to weak electrostatic interactions with the functional groups of AuNPs.

It is well worth mentioning that according to the experimental data^38^, the functionalized AuNPs show a considerable sensitivity against Pb^2+^, after Cd^2+^. The calculated EIE values confirm that these gold nanoparticles have remarkable electrostatic interactions with Pb^2+^. Moreover, the values of EIE for the interaction of the functionalized gold nanoparticles with metal ions show that the affinity of these nanoparticles against Cd^2+^ is as follows: MNA-CYS-AuNPs > MNA-AuNPs > CYS-AuNPs. This result is according to the sensitivity of the functionalized AuNPs based on the experimental results^38^.

To analyze the effect of metal ions on the interactions between water and functionalized AuNPs, the average number of the H-bonds between the functional groups of nanoparticles and water molecules in the presence and absence of metal ions were calculated and reported in Fig. 6-c. According to Fig. 6-c, H-bond interactions between water molecules and functional groups of AuNPs reduce significantly in the presence of Cd^2+^ in comparison with other metal ions. In other words, water molecules and metal ions compete with each other for interaction with MNA and CYS groups of AuNPs. Due to the considerable interactions of Cd^2+^ with the functionalized AuNPs, the possibility of the H-bond formation between these nanoparticles and water molecules decreases. Moreover, the functionalized AuNPs aggregate in the presence of Cd^2+^, which reduces the solvent accessible surface area (SASA) of gold nanoparticles as well as the possibility of the H-bond formation.

SASA is one of the parameters that can clearly confirm the aggregation process of the functionalized AuNPs. Figure 6-d shows the calculated average values of SASA for the functionalized AuNPs in the presence and absence of different metal ions. According to this figure, functionalized AuNPs due to the aggregation have smaller SASA in the presence of Cd^2+^ than that of without metal ions. Moreover, the minimum value of SASA for MNA-CYS-AuNPs shows the maximum aggregation for these nanoparticles against Cd^2+^. Therefore, MNA-CYS-AuNPs have the greatest interaction with this ion in comparison with other functionalized AuNPs. The interesting point is that the obtained magnitude of SASA for the functionalized gold nanoparticles is according to their sensitivity against Cd^2+^ based on the experimental data^38^.

**Figure 7:**
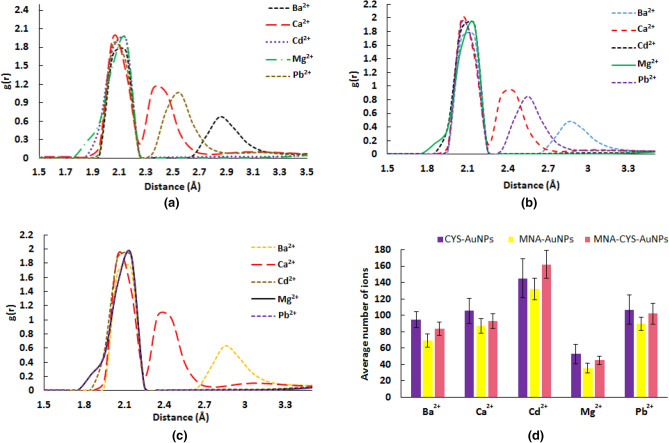
The calculated RDF plots of the O^….^metal ion (O atoms of CYS or MNA) pairs for the CYS-AuNPs (**a**), MNA-AuNPs (**b**) and MNA-CYS-AuNPs (**c**) and the calculated average number of different ions (**d**) around the functionalized AuNPs. The average values were calculated during 50 ns MD simulations and the error bars show the calculated standard deviation of the corresponding quantities.

RDF plots of the O^….^metal ion (O atoms of MNA or CYS) pairs (Fig. 7) show ion distribution around the functionalized AuNPs. The first sharp peak of the metal ions around MNA-AuNPs, CYS-AuNPs and MNA-CYS-AuNPs is in the range of 2.0–2.2 Å, which confirms that the ion distribution around the nanoparticles is similar to each other, but the functionalized AuNPs can only detect Cd^2+^. The calculated RDFs for Cd^2+^ and other metal ions show one and two ion shells around the functionalized AuNPs, respectively. The formation of one ion shell around the functionalized nanoparticles confirms the aggregation process. In other words, due to the strong interactions between the functional groups and Cd^2+^, this ion lies at a certain distance relative to the functionalized AuNPs (uniform distribution). The calculated number of the metal ions around the functionalized AuNPs in the range of 2.0–10.0 Å reveals that these nanoparticles have stronger interaction with Cd^2+^ than other metal ions. According to Fig. 7-d, the calculated average number of Cd^2+^ around the CYS-AuNPs, MNA-AuNPs, and MNA-CYS-AuNPs are 24, 22 and 27, respectively. These values confirm that each functional group can adsorb one Cd^2+^ (theoretical ratio 30 ions per functionalized AuNPs) confirming a considerable ability of the corresponding functionalized AuNPs for detection of Cd^2+^, especially MNA-CYS-AuNPs. Overall, based on the obtained results from MD simulations the functionalized AuNPs have more sensitivity against Cd^2+^ due to the stronger electrostatic interactions with this ion than other metal ions, which is in agreement with the experimental observations^38^.

### Quantum chemistry aspects of metal ion interactions with functional groups

MD simulation results reveal that Cd^2+^, due to the strong interactions, can aggregate the functionalized AuNPs in comparison with other metal ions. Therefore, DFT-D3 calculations at M06-2X-D3/6-311++G(d,p) level of theory were applied to investigate the nature and strength of the interactions between the metal ions and functional groups (MNA and CYS) from the quantum chemistry viewpoint. The optimized structures of the CYS-ion-CYS, MNA-ion-MNA and MNA-ion-CYS complexes in water are depicted in Figs. S2, S3, and S4, respectively. According to the optimized structures, metal ions make a bridge between the functional groups, which facilitates the aggregation of the nanoparticles. Therefore, the strength of the interactions of the metal ions and functional groups have a key role in the aggregation of the functionalized AuNPs.

Thermodynamic parameters of the MNA/CYS complexes with different metal ions such as binding energy (ΔE_bin_) and Gibbs energy of binding (ΔG_bin_) were calculated and reported in Table 1. According to the calculated ΔE_bin_ and ΔG_bin_ values, the complexes of Ba^2+^ and functional groups have the minimum stability in comparison with other metal ion complexes. Moreover, ΔG_bin_ values reveal that MNA-ion-CYS complexes are thermodynamically more stable than CYS-ion-CYS and MNA-ion-MNA complexes. Based on the calculated thermodynamic parameters, MNA and CYS show a greater reactivity against Cu^2+^, Mg^2+^, Pb^2+^, and Ca^2+^ than Cd^2+^, which is in contrast with the experimental and MD simulation results. The reported data^37^ for the functionalized silver nanoparticles with CYS groups by Venditti et al. revealed that CYS forms more stable complexes with Cu^2+^ and Hg^2+^ than Cd^2+^, which is approximately in agreement with the calculated thermodynamic parameters in this study. It is well worth mentioning that this discrepancy between experimental results and the DFT-D3 calculations is due to omitting the role of nanoparticles, counterions and water molecules that have crucial effects on the calculated results. Furthermore, the calculated thermodynamic parameters depend on the ratio of the electric charge to the metal ionic radii (ionic potential, Table 1), which means that metal ions having greater ionic potential (I = Z/r) form complexes with larger binding energy^60^. In this context, to compare the nature and strength of the metal ion interactions with CYS and MNA groups, 2D and 3D NCI analyses were employed.

**Table 1:** The calculated thermodynamic parameters, quantum chemistry reactivity indices, ionic potential and stabilization energies of the functional group (MNA and CYS) complexes with different metal ions in water.

	− ∆G_bin_ (kcal mol^−1^)	− ∆E_bin_ (kcal mol^−1^)	− E_HOMO_ (eV)	− E_LUMO_ (eV)	∆E_(LUMO–HOMO)_ (eV)	− µ (eV)	∑E(2) (kcal mol^−1^)
**CYS**							
Ba^2+^	1.53	17.49	7.35	1.34	6.01	4.34	11.64
Ca^2+^	29.10	46.91	7.43	1.36	6.07	4.39	46.16
Cd^2+^	22.43	41.07	7.32	1.40	5.92	4.36	154.18
Mg^2+^	55.04	73.89	7.49	1.38	6.11	4.43	102.78
Pb^2+^	85.93	157.43	7.35	1.39	5.96	4.37	99.30
Cu^2+^	134.92	150.92	7.39	1.79	5.60	5.59	82.11
Hg^2+^	1.55	20.92	7.42	1.36	6.06	4.39	69.25
Receptor	…	…	7.21	1.20	6.01	4.20	…
**MNA**							
Ba^2+^	0.73	17.06	7.21	1.40	5.81	4.30	9.85
Ca^2+^	26.13	45.92	7.29	1.42	5.87	4.35	46.76
Cd^2+^	19.73	39.35	7.18	1.48	5.70	4.33	158.81
Mg^2+^	53.33	71.83	7.36	1.43	5.93	4.39	102.43
Pb^2+^	85.77	102.59	7.40	1.46	5.94	4.43	97.33
Cu^2+^	129.28	143.69	7.24	1.85	5.39	4.54	79.89
Hg^2+^	− 0.79	18.93	7.27	1.41	5.86	4.34	70.76
Receptor	…	…	7.12	1.37	5.75	4.24	…
**MNA-CYS**							
Ba^2+^	8.17	13.80	7.21	1.41	5.80	4.31	13.95
Ca^2+^	35.98	43.82	7.29	1.42	5.87	4.35	46.25
Cd^2+^	24.77	37.04	7.10	1.47	5.63	4.28	155.79
Mg^2+^	60.84	69.35	7.37	1.43	5.94	4.40	99.52
Pb^2+^	94.35	102.04	7.37	1.44	5.93	4.40	91.77
Cu^2+^	140.39	146.59	7.24	1.79	4.45	4.51	84.48
Hg^2+^	6.21	15.40	7.25	1.41	5.84	4.33	61.61
Receptor	…	…	7.03	1.35	5.68	4.19	…

I = 1.48, 2.00, 2.10, 2.77, 1.68, 2.73 and 1.96 for Ba^2+^, Ca^2+^, Cd^2+^, Mg^2+^, Pb^2+^, Cu^2+^ and Hg^2+^ respectively.

By employing NCI analysis, it is possible to determine the nature of the interactions (repulsive or attractive), based on the electron density (ρ) and the sign of the second derivative in the perpendicular of the bond (λ_2_). The negative and positive characters of λ_2_ show the bonding interactions (for example, H-bond or strong electrostatic interaction) and nonbonding interactions (such as strict repulsion), respectively. Moreover, the negligible values of λ_2_ (λ_2_ ≈ 0) confirm the vdW interactions.

**Figure 8:**
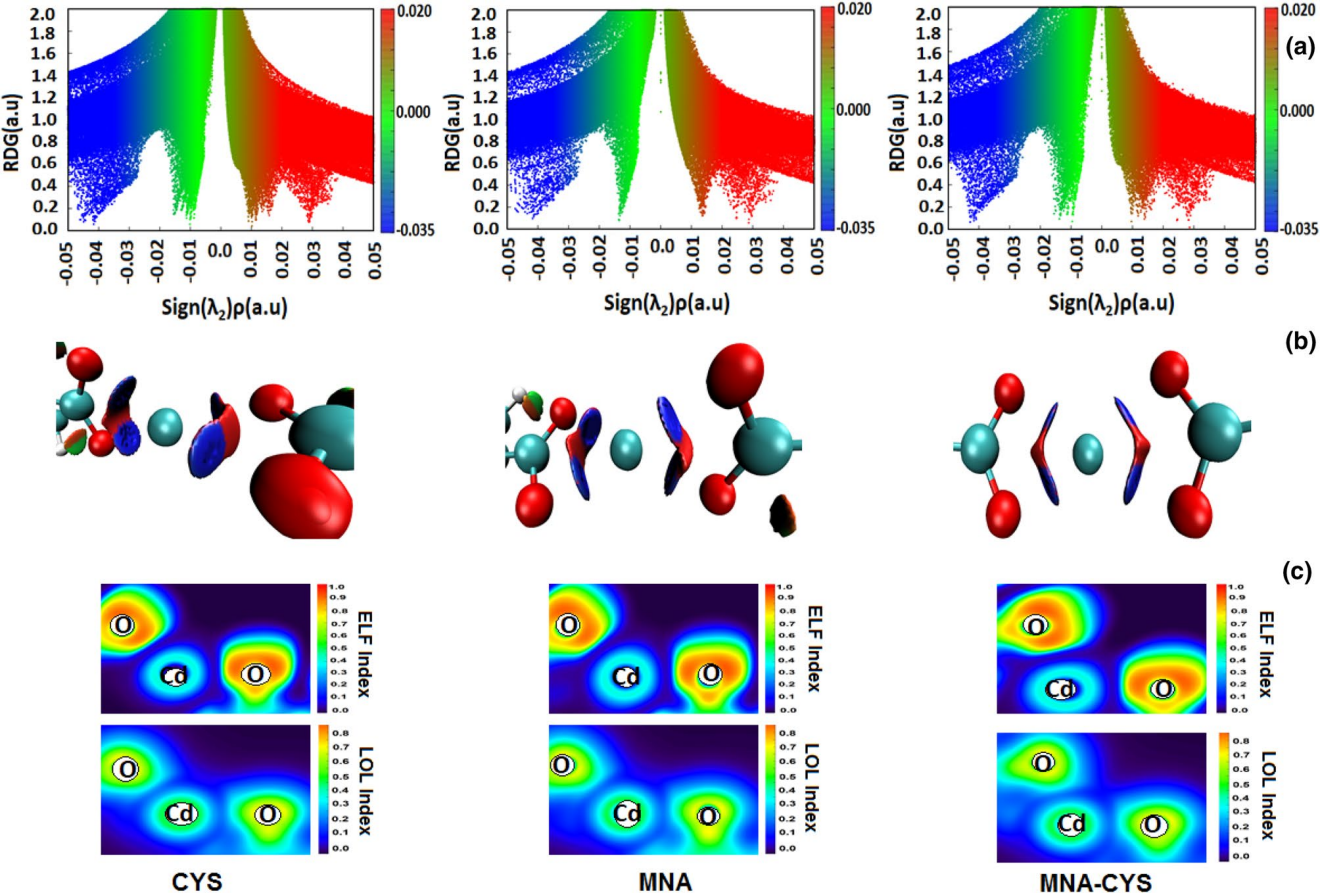
The 2D (**a**) and 3D (**b**) NCI plots and ELF and LOL graphs (**c**) of the Cd^2+^ complexes with MNA and CYS groups.

Figure 8-a shows the 2D NCI plots of the reduced density gradient (RDG) versus the electron density for Cd^2+^ complexes with MNA and CYS groups (2D NCI plots of other metal ion complexes are represented in Fig. S5). In the 2D NCI plot, three definite regions are specified by blue, green, and red colors, which reveal bonding, vdW, and repulsive interactions, respectively. According to Fig. 8-a, the negative values of sign (λ_2_)ρ confirm strong electrostatic interaction between Cd^2+^ and the corresponding functional groups. The 2D NCI plot of Ba^2+^ complexes indicates that this metal ion has only vdW interactions with MNA and CYS (Fig. S5) that is weaker than Cd^2+^ interactions with these groups. This result confirms the MD simulation data and reveals that Ba^2+^ due to weak interactions with functional groups cannot eliminate the repulsive interactions of the functionalized AuNPs. Moreover, on the basis of sign (λ_2_)ρ values, Cd^2+^ and Pb^2+^ have stronger electrostatic interactions with MNA and CYS groups in comparison with Mg^2+^ and Ca^2+^, which is according to the MD simulation results.

Figure 8-b shows the 3D NCI plots of Cd^2+^ interactions with functional groups. In this figure, each interaction is represented by color-filled isosurfaces such as, red, blue and green, which reveal nonbonding, bonding and vdW interactions, respectively. The comparison between the obtained 3D NCI plots clearly shows that Cd^2+^ and Pb^2+^ (Fig. S6) have considerable interactions with CYS and MNA groups. According to Fig. S6, the green isosurfaces between O atoms of the functional groups (COO^−^) and Ba^2+^ confirm vdW interaction, which is significantly weaker than electrostatic interactions. Also, the dark blue isosurfaces in the region between Cd^2+^ and the corresponding receptors show bonding or strong electrostatic interactions. This result indicates that MNA and CYS have greater sensitivity against Cd^2+^ in comparison with other metal ions. NCI analysis reveals that metal ions have electrostatic interactions with the functional groups and Cd^2+^ has the highest interaction with MNA and CYS.

### Reactivity indices and donor–acceptor interactions

To study the electronic properties of MNA and CYS during the complexation with different metal ions, by employing HOMO–LUMO analysis, quantum chemistry reactivity indices such as electronic chemical potential (µ) and band gap (ΔE_LUMO–HOMO_) were calculated. Comparison between the HOMO and LUMO energy levels of CYS and MNA in the presence and absence of different metal ions indicates a remarkable charge transfer and interaction between these receptors and metal ions (Table 1). According to Table 1, the band gap is reduced in the presence of Cd^2+^ in comparison with other metal ions, which confirms the sensitivity of MNA and CYS against Cd^2+^ and a conductivity enhancement that is a key factor in ion sensing.

According to the experimental results, the functionalized AuNPs with MNA groups have more sensitivity against Cd^2+^ in comparison with CYS-AuNPs^34^. The calculated band gap values reveal that MNA, due to a smaller band gap, has a greater reactivity against different metal ions than CYS. Therefore, a higher sensitivity of the functionalized AuNPs with MNA groups against Cd^2+^ is predictable. The calculated negative values of µ reveal that MNA and CYS as the receptors can form stable complexes with Cd^2+^, which is according to the MD simulation and NCI results.

To investigate the charge transfer and donor–acceptor interactions between CYS/MNA receptors and different metal ions, NBO analysis was applied. Based on the stabilization energy, E(2), values the main interactions are related to the antibonding orbital (LP*) of the metal ions as the acceptor and lone pair electrons of O atoms of the COO^−^ groups as the donor. According to the ∑E(2) values in Table 1, the minimum and maximum donor–acceptor interactions are related to the Ba^2+^ and Cd^2+^ with functional groups, respectively, according to the EIE results from the MD simulations. This means that Ba^2+^ has weak interactions with functional groups, which is not sufficient enough to overcome the repulsive interactions between the MNA/CYS groups on the AuNPs surface to facilitate their aggregation. NBO analysis indicates that the aggregation of the functionalized AuNPs in the presence of Cd^2+^ is due to the remarkable molecular orbital and donor–acceptor interactions of this ion with MNA/CYS groups.

To show the electrostatic interactions between metal ions and receptors (MNA and CYS), ELF and LOL analyses were applied (Figs. 8-c, S7, S8 and S9). ELF and LOL reveal a similar definition because they are related to the kinetic energy density. LOL indicates the gradients of the localized orbitals, while ELF reveals the electron pair density. Large and small values of these parameters reveal the covalent and electrostatic interactions, respectively. According to the ELF and LOL graphs (Figs. 8-c, S7, S8 and S9), high values of ELF and LOL around the O atoms show the highly localized bonding and nonbonding electrons. The blue region between Cd^2+^ and O atoms of the receptor groups (COO^−^) indicates the localized electrons and confirms the considerable electrostatic interaction. According to ELF and LOL plots, Cd^2+^ and Pb^2+^ have strong electrostatic interactions with MNA and CYS groups, through which can form the most stable complexes with the functional groups.

## Conclusion

MD simulations indicate that MNA and CYS as the functional groups can increase the stability of AuNPs in water and their resistance against aggregation. The vdW interactions between the AuNPs elevates the aggregation process, while negatively charged functional groups on the surface of AuNPs increase the repulsive interactions of the gold nanoparticles, which prevent the nanoparticle aggregation. MNA-AuNPs show more stability in water in comparison with CYS-AuNPs and MNA-CYS-AuNPs, which confirms the efficient role of the functional groups on the stability of AuNPs. The functionalized AuNPs have considerable sensitivity against Cd^2+^ in comparison with other metal ions. Cd^2+^ due to the strong electrostatic interactions with MNA and CYS groups of AuNPs can eliminate the repulsive interactions of functionalized AuNPs and induces them to aggregation. MD simulation results show that MNA-CYS-AuNPs aggregate in the presence of Cd^2+^ more than other functionalized AuNPs, confirming higher sensitivity for this nanoparticle.

NCI analysis reveals that Ba^2+^ and Cd^2+^ have the minimum and maximum electrostatic interactions with MNA and CYS groups, respectively. Quantum chemistry calculations indicate a greater response in the theoretical band gap of MNA and CYS in the presence of Cd^2+^. Moreover, molecular orbital interactions between the MNA and CYS and different metal ions can be a driving force for the functionalized AuNPs aggregation. Overall, on the basis of the MD simulations and DFT-D3 calculations, MNA-CYS-AuNP is a better sensor for the detection of Cd^2+^ due to the stronger electrostatic and molecular orbital interactions with this ion in comparison with other functionalized AuNPs.

## Supplementary Information

Supplementary Information.
