# Supplementary material for: A theoretical approach on the ability of functionalized gold nanoparticles for detection of Cd2+
Source: Sci Rep. 2021 Dec 6;11:23422. doi: 10.1038/s41598-021-02933-5 (PMC8648727; doi:10.1038/s41598-021-02933-5)
Supplement: Supplementary file 1 — Supplementary Information. [file 41598_2021_2933_MOESM1_ESM.docx]

**Supporting information**

**A Theoretical Approach on the Ability of Functionalized Gold Nanoparticles for Detection of Cd^2+^**

Mohammad Khavani*^a^, Aliyeh Mehranfar^b^, Mohammad Izadyar*^b^

^a^ Department of Chemistry and Materials Science, School of Chemical Engineering, Aalto

University, P.O. Box 16100, FI-00076 Aalto, Finland

^b^ Research Center for Modeling and Computational Sciences, Faculty of Science, Ferdowsi University of Mashhad, Mashhad, Iran

Mohammad.khavanisariani@aalto.fi

[izadyar@um.ac.ir](mailto:izadyar@um.ac.ir)


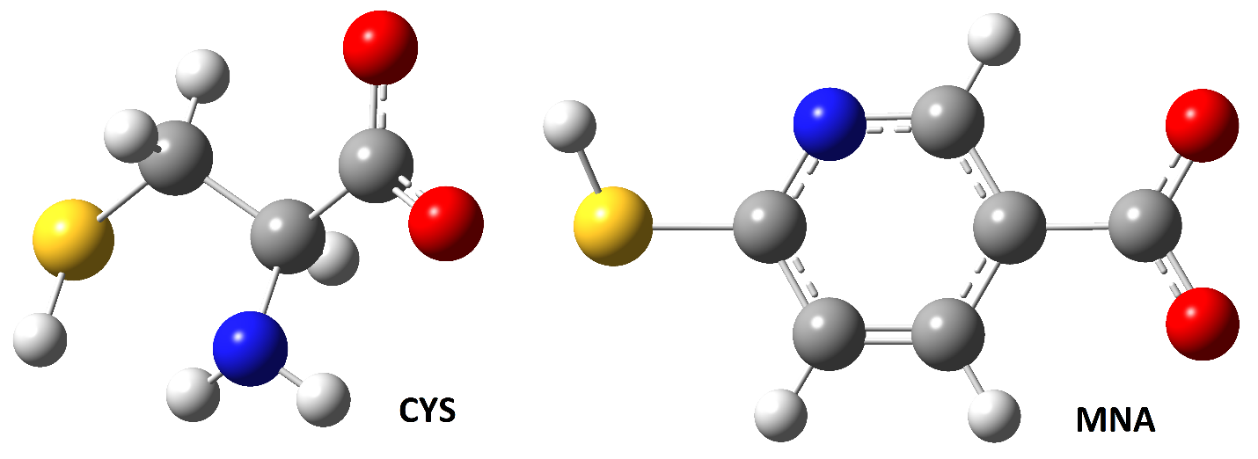


Figure S1. The structure of the MNA and CYS functional groups.

**
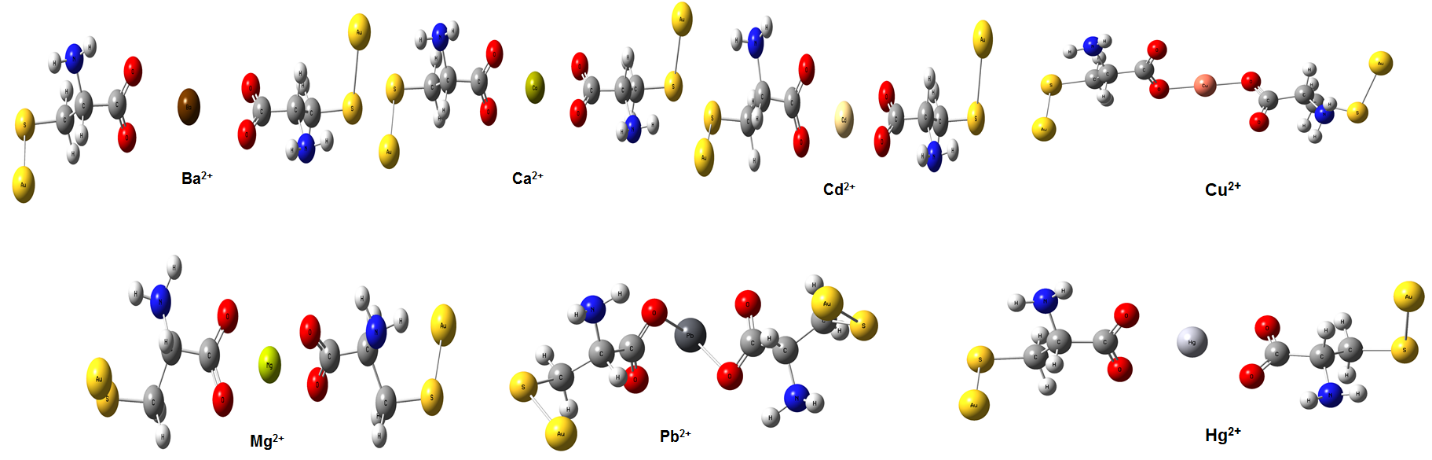
**

Figure S2. The optimized structures of CYS-ion-CYS complexes at M06-2X-D3/6-311++G(d,p) level of theory in water.


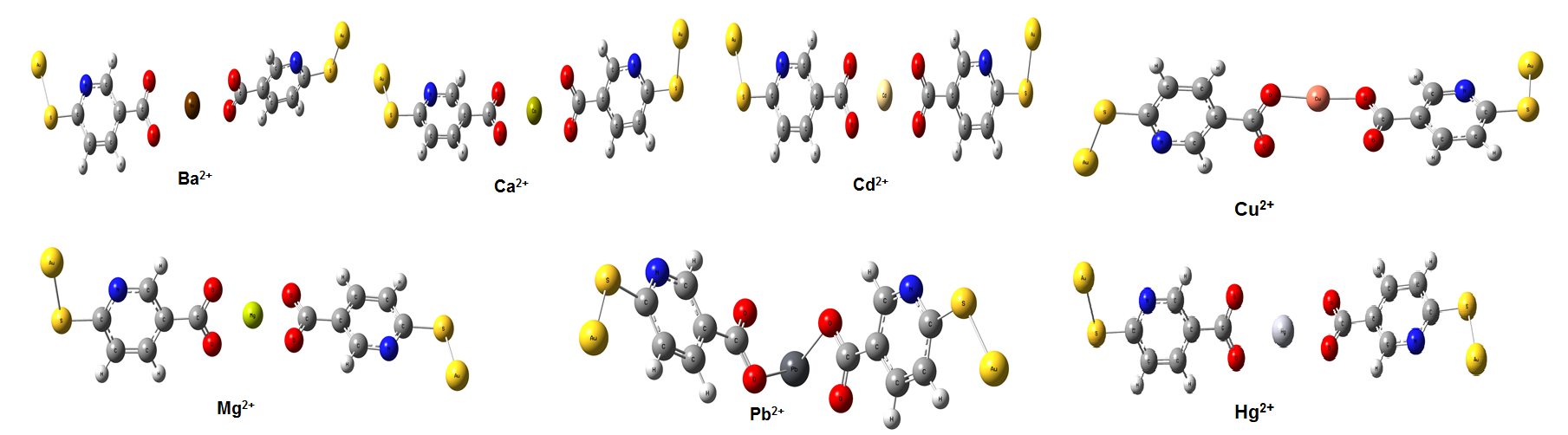


Figure S3. The optimized structures of MNA-ion-MNA complexes at M06-2X-D3/6-311++G(d,p) level of theory in water.


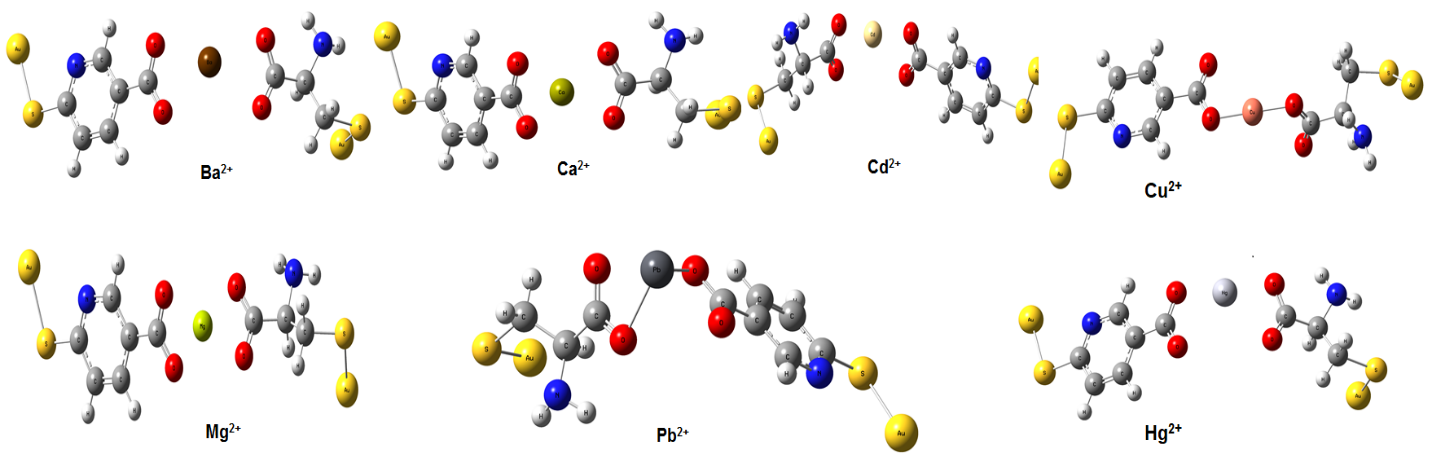


Figure S4. The optimized structures of MNA-ion-CYS complexes at M06-2X-D3/6-311++G(d,p) level of theory in water.


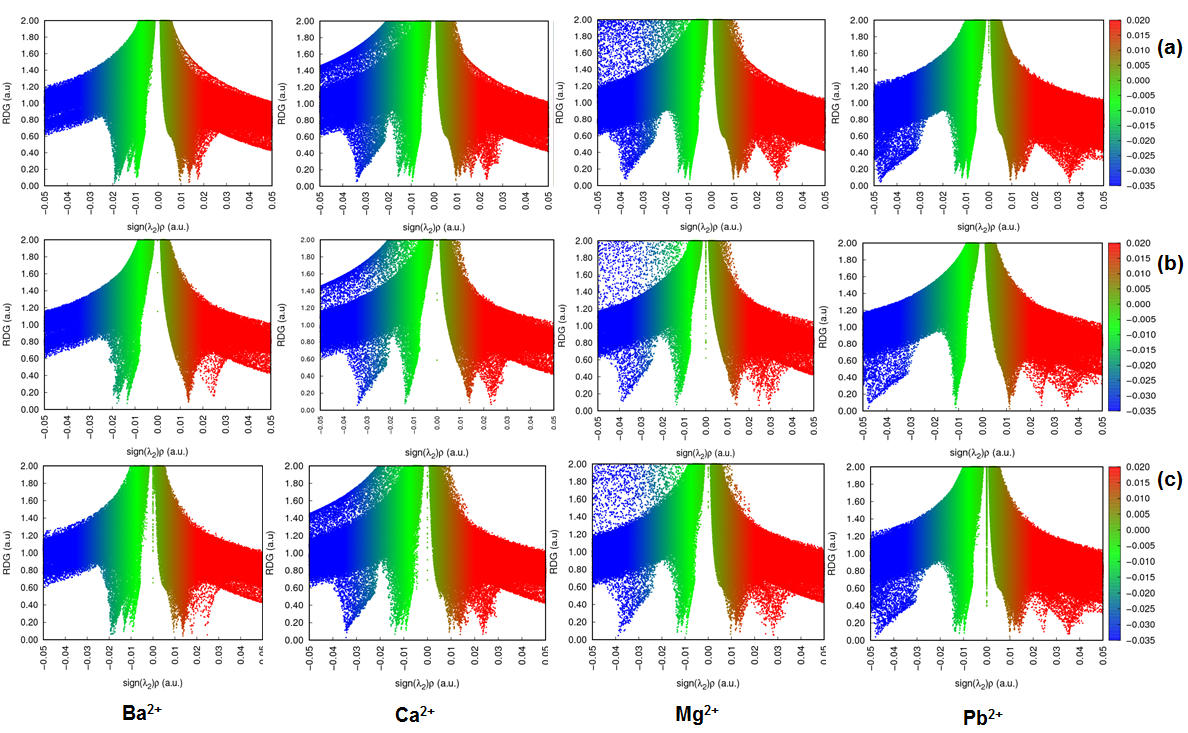


Figure S5. The 2D NCI plots of CYS-ion-CYS (a), MNA-ion-MNA (b) and MNA-ion-CYS (c) complexes at M06-2X-D3/6-311++G(d,p) level of theory in water.


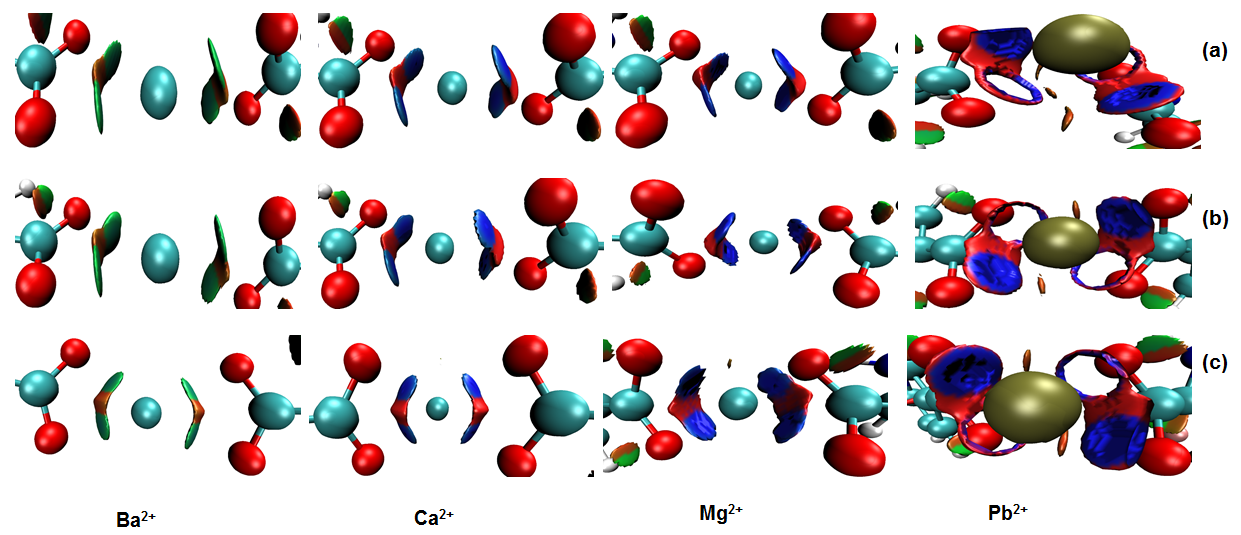


Figure S6. The 3D NCI plots of CYS-ion-CYS (a), MNA-ion-MNA (b) and MNA-ion-CYS (c) complexes at M06-2X-D3/6-311++G(d,p) level of theory in water.


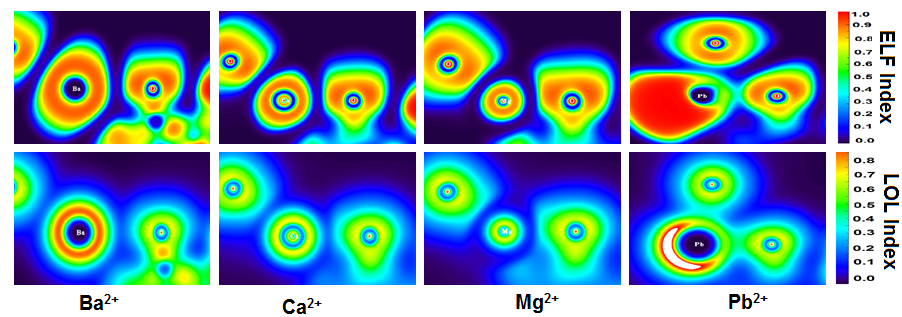


Figure S7. The ELF and LOL plots of CYS-ion-CYS complexes at M06-2X-D3/6-311++G(d,p) level of theory in water.


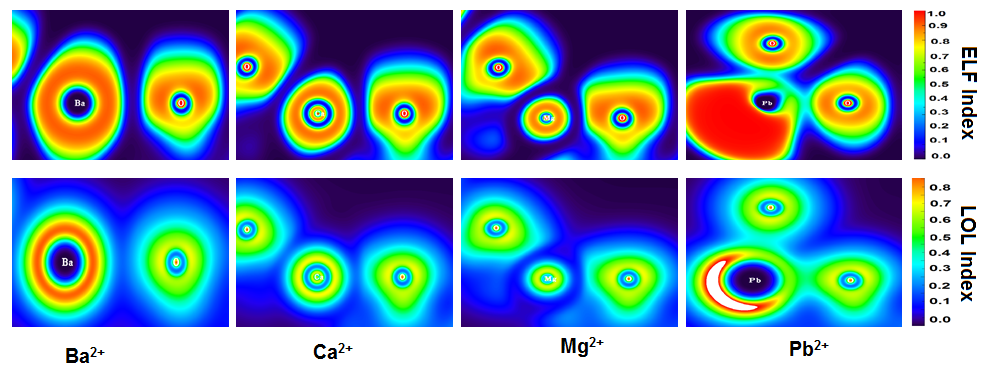


Figure S8. The ELF and LOL plots of MNA-ion-MNA complexes at M06-2X-D3/6-311++G(d,p) level of theory in water.


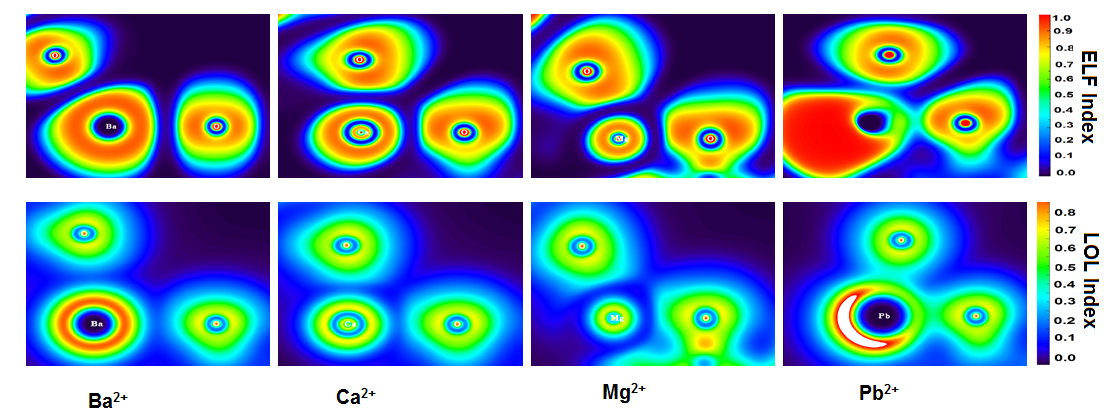


Figure S9. The ELF and LOL plots of CYS-ion-MNA complexes at M06-2X-D3/6-311++G(d,p) level of theory in water.

**Optimized XYZ coordinates of the functional group complexes with different metal ions.**

**CYS-Cu**

S 1.12108800 4.43357300 -1.10766600

C 1.75915400 2.81217600 -0.53746800

C 2.85118700 2.90958400 0.52326400

H 0.93831400 2.20281600 -0.16850300

H 2.17062000 2.34390300 -1.43644700

H 2.39014200 3.35256100 1.42339100

N 3.98243000 3.68625100 0.03463200

C 3.32040900 1.51586500 0.96982900

H 3.72165100 4.66570000 -0.01707600

H 4.75399900 3.59564400 0.68689600

O 4.51859900 1.30957800 1.17676000

O 2.38401100 0.66011000 1.14168600

Au 0.35068200 5.38551400 0.85661500

S 3.05058500 -7.10322600 4.26844800

C 2.82604900 -5.62142500 3.21301100

C 3.80821600 -4.62136100 3.82078800

H 2.99809500 -5.84759900 2.16416300

H 1.79028600 -5.30934100 3.36129600

H 4.80066800 -4.78935900 3.39900500

N 3.84082900 -4.96553300 5.24170500

C 3.38032400 -3.17524900 3.53597900

H 4.73385100 -4.81030400 5.69576300

H 3.10152200 -4.50033400 5.76297500

O 2.98702200 -2.46800000 4.46152700

O 3.45138900 -2.86166600 2.30268500

Au 5.16102000 -7.89102400 3.69378200

Cu 2.94472800 -1.07717400 1.76687700

CYS-Hg

S 1.38420300 5.16344300 -0.80441100

C 1.52646300 3.37849000 -0.41359100

C 2.74128200 3.02604000 0.43674600

H 0.61936700 3.03415600 0.07628800

H 1.61004300 2.88390700 -1.38546800

H 2.61004800 3.52213100 1.41470900

N 3.97594000 3.42258700 -0.22767500

C 2.76967600 1.52942700 0.77220700

H 4.02715800 4.43619900 -0.25648200

H 4.76678600 3.08076000 0.30758700

O 3.89324200 0.97636200 0.91808200

O 1.68182200 0.93623100 0.94550900

Au 1.20886800 6.12055400 1.29619000

S 3.75754900 -7.59913600 4.26168400

C 3.28622900 -6.08681600 3.33967000

C 3.70781300 -4.79132700 4.02293200

H 3.69585400 -6.12449300 2.33352800

H 2.19492400 -6.12414500 3.27804000

H 4.81186400 -4.78381500 4.05056200

N 3.11671300 -4.68437800 5.35002300

C 3.34750300 -3.56647700 3.17268400

H 3.54191400 -5.37542700 5.96030700

H 3.31377700 -3.76393900 5.72802800

O 3.01109400 -2.50870900 3.75879900

O 3.47215200 -3.65749400 1.92590200

Au 6.06352900 -7.41657700 4.36667500

Hg 3.00105600 -1.19587900 1.60539200

MNA-Cu

S -8.53469200 3.99977200 -0.80812600

C -7.62111900 2.57485100 -1.32110800

C -7.93831700 1.34826400 -0.71070600

N -6.67138900 2.68157800 -2.25441100

C -7.24049500 0.21791600 -1.08796600

H -8.71528300 1.30064600 0.04161200

C -6.00874800 1.57579400 -2.60560900

C -6.24377900 0.31915600 -2.06153200

H -7.45423100 -0.74468800 -0.63950800

H -5.24313300 1.69184600 -3.36547200

C -5.44170600 -0.87153000 -2.51567200

O -5.74184100 -1.97815100 -1.94694700

O -4.56982600 -0.72554700 -3.37820800

Au -7.57807400 5.69995000 -2.07827400

S -0.62251300 -10.53740700 -4.95606800

C -1.61046800 -9.15431400 -4.66782500

C -2.66095000 -8.90840500 -5.57362100

N -1.34696700 -8.36985000 -3.60220300

C -3.46606200 -7.80021800 -5.35675300

H -2.82471300 -9.57346200 -6.41148300

C -2.12511700 -7.32259700 -3.41613900

C -3.20310200 -6.98830500 -4.26428300

H -4.28808100 -7.56139400 -6.01937000

H -1.91334400 -6.69255800 -2.56010300

C -4.05858000 -5.76496500 -3.98628600

O -4.98905500 -5.51747700 -4.75044400

O -3.71093200 -5.10147700 -2.95648500

Au 0.91442000 -10.46584300 -3.19191700

Cu -4.72575000 -3.51610700 -2.51612000

MNA-Hg

S -8.33047800 4.43871100 -0.88810400

C -7.64432100 2.87479300 -1.34382300

C -8.10885800 1.74120500 -0.65191900

N -6.72177600 2.79378700 -2.30653800

C -7.58280200 0.50649000 -0.97233200

H -8.86045800 1.84574800 0.12012300

C -6.23325400 1.58762100 -2.60999700

C -6.61854200 0.41165500 -1.97916200

H -7.90675800 -0.38924000 -0.45629500

H -5.48628600 1.55204700 -3.39602800

C -6.00320700 -0.90626200 -2.35121300

O -6.28684800 -1.90127000 -1.63126700

O -5.23675900 -0.96353800 -3.34184100

Au -7.19363500 5.92516400 -2.27166400

S -0.55626600 -10.77666000 -5.40326700

C -1.46111900 -9.35115600 -4.88011500

C -2.54760600 -8.94609700 -5.67698900

N -1.10818000 -8.69656000 -3.77034900

C -3.27504500 -7.83778000 -5.29361100

H -2.80129600 -9.49992100 -6.57185900

C -1.81780900 -7.61927200 -3.42242300

C -2.90924400 -7.14323100 -4.13789600

H -4.12007900 -7.49477300 -5.87861500

H -1.50400500 -7.10414200 -2.52076400

C -3.65689100 -5.91852500 -3.69654700

O -4.70425400 -5.60869200 -4.30662600

O -3.18469800 -5.24903300 -2.73474700

Au 1.04879400 -10.99988800 -3.73241600

Hg -4.82216600 -3.45570700 -2.85474500

MNA-CYS-Cu

S 1.68133900 -8.25084600 3.75434900

C 1.05309300 -7.15167500 2.42916400

C 2.18909800 -6.14457000 2.25565400

H 0.79905200 -7.71068400 1.53260300

H 0.15790500 -6.67996000 2.83937000

H 2.93359700 -6.55272800 1.56989100

N 2.78610100 -6.04078900 3.58677100

C 1.66699500 -4.81246900 1.70077400

H 3.78581400 -5.87270000 3.59112000

H 2.31506500 -5.34514900 4.16041900

O 1.66822700 -3.81626000 2.42151500

O 1.23212600 -4.89682000 0.50571800

Au 3.40211100 -9.43389600 2.73590900

S -3.63258100 3.24819700 -3.96665300

C -2.98982200 1.82506700 -3.13656200

C -3.91215500 0.98058500 -2.49234900

N -1.67715200 1.57961900 -3.13421300

C -3.44109500 -0.13942700 -1.83729500

H -4.96930800 1.21262500 -2.51551300

C -1.24177800 0.48979100 -2.49426900

C -2.07002300 -0.40620100 -1.82949500

H -4.11894700 -0.81527700 -1.32941700

H -0.17133600 0.31649900 -2.50989200

C -1.51905400 -1.61889800 -1.12743400

O -2.29595100 -2.38783800 -0.55292300

O -0.24800700 -1.75884400 -1.18624100

Au -1.71484200 4.24680100 -4.82869800

Cu 0.45890800 -3.31565000 -0.29126500

MNA-CYS-Hg

S 2.23058700 -8.60658900 2.19435600

C 1.13652300 -7.14991300 2.39812400

C 1.80624400 -5.81910600 2.08004000

H 0.24425200 -7.27071300 1.78902600

H 0.84587800 -7.16559200 3.45233200

H 2.03860900 -5.81794500 1.00030700

N 2.99334700 -5.62048000 2.90331900

C 0.83778400 -4.64253400 2.26760100

H 3.71311700 -6.27655500 2.61692200

H 3.34297700 -4.67984400 2.75552900

O 1.33469300 -3.53219300 2.59162700

O -0.38324000 -4.81630000 2.04665800

Au 2.90191900 -8.43415200 -0.01424500

S -2.71080500 1.90084900 -5.11436800

C -2.40361500 1.03991400 -3.60068000

C -3.18484300 -0.09939400 -3.33500200

N -1.46205400 1.47058400 -2.75721300

C -2.97134400 -0.79065000 -2.15957800

H -3.93768500 -0.41964500 -4.04393900

C -1.26700200 0.78347000 -1.62738100

C -1.98722000 -0.34959400 -1.27132800

H -3.55324800 -1.67167000 -1.91627700

H -0.49406500 1.15520400 -0.96327900

C -1.72073700 -1.07733000 0.01933200

O -2.45317700 -2.04164800 0.31342200

O -0.75795700 -0.66703500 0.73165100

Au -1.18925400 3.65949000 -5.01505000

Hg -0.82230600 -2.31212400 2.50913000

CYS-Ba

S 1.35536400 5.42488700 -1.22022000

C 1.58044500 3.66594300 -0.75217900

C 2.65917700 3.43356800 0.29885900

H 0.63484000 3.25222800 -0.41110000

H 1.86962100 3.16636300 -1.68170600

H 2.32753800 3.94387500 1.22040800

N 3.95274100 3.91722300 -0.16693800

C 2.74489500 1.94401900 0.68468900

H 3.96984300 4.93129300 -0.13715500

H 4.67445000 3.56495400 0.45256200

O 3.88123800 1.45329800 0.89614800

O 1.66329500 1.31700300 0.81267900

Au 0.78094800 6.40288800 0.79672000

S 3.61434700 -7.99413600 4.37267500

C 3.17262100 -6.40911500 3.56085400

C 3.82121700 -5.18221700 4.19027000

H 3.41669300 -6.45641300 2.50256200

H 2.08613100 -6.33671200 3.66909300

H 4.91232100 -5.28480800 4.05328900

N 3.44756100 -5.05915000 5.59340800

C 3.45056400 -3.90172500 3.41673600

H 3.94056400 -5.75614200 6.14156000

H 3.71777700 -4.13907600 5.92378600

O 3.24265600 -2.85367600 4.07514200

O 3.42726200 -3.97140300 2.16197100

Au 5.90947100 -8.07629300 4.08833700

Ba 2.88328900 -1.16499800 1.69146600

CYS-Ca

S 1.33559800 4.82269000 -1.12647100

C 1.59272400 3.11119300 -0.52413600

C 2.70836300 2.98203300 0.50803400

H 0.66392200 2.71823600 -0.11815900

H 1.86006500 2.53624700 -1.41553800

H 2.40386600 3.56901300 1.39211000

N 3.98220800 3.41931900 -0.04495100

C 2.81008900 1.54435000 1.01897600

H 3.96569300 4.42681200 -0.16650600

H 4.72710300 3.18996600 0.60399200

O 3.94711900 1.04120300 1.22016200

O 1.74282900 0.92125400 1.26898000

Au 0.74974400 5.93970300 0.81500800

S 3.47491300 -7.52530300 4.03030900

C 3.07039000 -5.89219800 3.30449900

C 3.79937000 -4.72646300 3.96321400

H 3.27348700 -5.89988500 2.23644400

H 1.99352000 -5.77653400 3.45796300

H 4.87774000 -4.86495000 3.76782800

N 3.49152200 -4.65354500 5.38471100

C 3.45383400 -3.40344200 3.27860100

H 3.92317200 -5.43847600 5.86195200

H 3.87720500 -3.79724500 5.76791500

O 3.35538400 -2.36302200 3.98245000

O 3.32788600 -3.38223000 2.02448000

Au 5.76131400 -7.66842400 3.69653200

Ca 2.96780900 -0.99862700 2.04551900

CYS-mg

S 1.40153000 4.53988600 -0.92727500

C 1.67102100 2.80736000 -0.40158600

C 2.71332100 2.65756400 0.70407700

H 0.73031800 2.36362000 -0.08410400

H 2.02151800 2.28972900 -1.29901200

H 2.31285800 3.16402400 1.59987400

N 4.00404700 3.18016400 0.28446500

C 2.84337000 1.20309600 1.12042200

H 3.93073000 4.18680800 0.17427300

H 4.69903800 2.99293100 0.99932600

O 3.97669800 0.71258000 1.38482000

O 1.80513500 0.49259700 1.25091600

Au 0.70125900 5.55179000 1.03463600

S 3.33437400 -7.18016800 3.98408800

C 2.97244700 -5.56750200 3.19712600

C 3.78861100 -4.41149400 3.76782100

H 3.12753100 -5.63347100 2.12276000

H 1.91088100 -5.38955400 3.39009400

H 4.84700200 -4.60550500 3.51851000

N 3.56115300 -4.26001100 5.19691800

C 3.46375800 -3.11344200 3.04987800

H 3.94096700 -5.06938800 5.67766100

H 4.04715800 -3.43628700 5.53498600

O 3.45868400 -2.02159600 3.68383900

O 3.24453500 -3.11695000 1.80348000

Au 5.60034600 -7.44152100 3.57993600

Mg 3.02857900 -1.06358300 1.88599000

CYS-Cd

S 1.37284500 4.87562700 -0.98555900

C 1.53196100 3.13389900 -0.43765600

C 2.70978800 2.88974500 0.49672500

H 0.60931500 2.81011400 0.03777000

H 1.67363600 2.56087600 -1.35833200

H 2.52016300 3.47223900 1.41645500

N 3.97110600 3.24723500 -0.13875100

C 2.75851600 1.44222800 0.98187000

H 4.00175200 4.25352400 -0.27012700

H 4.73745700 2.98720600 0.47297500

O 3.87275800 0.94298600 1.28179600

O 1.67937000 0.81027100 1.13822500

Au 1.19765000 6.01900400 1.02168900

S 3.65116100 -7.47039300 3.85357100

C 3.23989800 -5.82663900 3.15858300

C 3.77381300 -4.65416100 3.97660900

H 3.59824700 -5.75615000 2.13455500

H 2.14665100 -5.79201900 3.15133000

H 4.87599400 -4.69037300 3.92136400

N 3.27548400 -4.70148900 5.34382500

C 3.39766800 -3.32601600 3.32262000

H 3.69551900 -5.49139900 5.82344500

H 3.54593400 -3.85411600 5.83106900

O 3.09464000 -2.34436700 4.04561700

O 3.44035700 -3.24103300 2.06567500

Au 5.96451700 -7.46334700 3.83705800

Cd 2.80066900 -1.00177300 2.11151500

CYS-Pb

S 3.06459700 4.39200300 0.47967700

C 2.01857800 2.89763800 0.62120400

C 2.81531800 1.60084300 0.73452100

H 1.34341700 2.99165700 1.46851500

H 1.42766800 2.87672200 -0.29886400

H 3.37265100 1.63981500 1.68717100

N 3.67874800 1.41094000 -0.42084300

C 1.88022300 0.41433500 0.89598800

H 4.39179600 2.13365600 -0.41818400

H 4.14381800 0.51158200 -0.35771000

O 2.17759700 -0.69178800 0.34493400

O 0.85160700 0.50935600 1.60744100

Au 4.25588400 4.35523300 2.46562200

S 5.13734200 -5.65668700 4.29470600

C 3.73988400 -4.97757500 3.32794200

C 3.54083800 -3.47643200 3.51234300

H 3.87072300 -5.20551400 2.27272800

H 2.85570100 -5.50633200 3.69477900

H 4.43326500 -2.97340400 3.09841300

N 3.29989600 -3.14236300 4.90824200

C 2.39497300 -2.97521400 2.64949300

H 4.13885000 -3.34860000 5.44180500

H 3.11104000 -2.14931000 4.99411400

O 1.69091500 -2.00107700 3.06488400

O 2.17743400 -3.47613600 1.52123700

Au 6.94096700 -4.47364700 3.45450900

Pb 0.38314000 -1.84802300 1.18924800

MNA-Mg

S -8.28573300 3.90251300 -0.85138200

C -7.51643500 2.39882900 -1.35421200

C -7.82165000 1.23404200 -0.62256500

N -6.67640800 2.38949200 -2.39524100

C -7.23628000 0.04316100 -0.99594400

H -8.50500600 1.28265200 0.21539600

C -6.11730900 1.22985800 -2.74130100

C -6.35832100 0.02944400 -2.08362700

H -7.44574900 -0.87589300 -0.46145900

H -5.44074000 1.25071100 -3.58928100

C -5.70072800 -1.22129600 -2.52916800

O -5.94487800 -2.31090600 -1.93347600

O -4.89182000 -1.20714400 -3.50352400

Au -7.46043500 5.42439200 -2.41436300

S -1.03330900 -10.58450900 -5.03614000

C -1.79877600 -9.04594100 -4.64756500

C -2.91843800 -8.66958100 -5.41558100

N -1.31965600 -8.28127800 -3.65989900

C -3.54109100 -7.47094000 -5.14076100

H -3.27574700 -9.31690100 -6.20587700

C -1.93424200 -7.12553700 -3.40717500

C -3.04315100 -6.66898000 -4.11000200

H -4.40409100 -7.14516700 -5.70958200

H -1.52478900 -6.52351300 -2.60270000

C -3.66303800 -5.36693500 -3.77139400

O -4.66518500 -4.95089700 -4.42306300

O -3.18970400 -4.66033300 -2.83196800

Au 0.71331600 -10.69583500 -3.49969100

Mg -4.63445300 -3.24803500 -3.23947900

MNA-Cd

S -8.40618500 4.17929800 -0.78208300

C -7.62970200 2.66996400 -1.26940300

C -8.12527500 1.47705800 -0.71044100

N -6.61298700 2.68307700 -2.13657700

C -7.54436400 0.28060000 -1.07669500

H -8.95254600 1.50710900 -0.01298700

C -6.06229800 1.51582500 -2.47731000

C -6.48256100 0.28635100 -1.98470700

H -7.90195900 -0.66032600 -0.67540800

H -5.24127100 1.55591700 -3.18546700

C -5.83594900 -0.98196900 -2.43535600

O -6.27346700 -2.07766900 -1.99877900

O -4.87778800 -0.92300300 -3.25555600

Au -7.29035700 5.76374300 -2.07076300

Cd -4.72091500 -3.23822700 -3.37163600

S -0.87316000 -10.87354600 -4.96662900

C -1.64156100 -9.31209500 -4.66544800

C -2.90360700 -9.09262000 -5.24848700

N -1.03433400 -8.39149900 -3.91098900

C -3.53872000 -7.88818600 -5.02570700

H -3.36170600 -9.86253100 -5.85584500

C -1.66407100 -7.23186300 -3.70755100

C -2.91287900 -6.92319600 -4.23294000

H -4.51285200 -7.68280700 -5.45367900

H -1.14925800 -6.50354100 -3.08996400

C -3.55824400 -5.60728600 -3.94360200

O -4.69627900 -5.36486500 -4.42586100

O -2.94821200 -4.77663200 -3.21761100

Au 1.11865800 -10.71160700 -3.77354300

MNA-Ca

S -8.28558800 4.28520900 -0.71532300

C -7.60212900 2.74422200 -1.24586900

C -7.99379000 1.58655400 -0.54789200

N -6.74413600 2.70453300 -2.26935700

C -7.47395600 0.36867300 -0.93693900

H -8.69190700 1.65993600 0.27620200

C -6.25125900 1.51708200 -2.62953400

C -6.57642400 0.31901500 -2.00586800

H -7.75018500 -0.54658800 -0.42674300

H -5.55736000 1.51336800 -3.46350900

C -5.97697600 -0.97147400 -2.46867700

O -6.30997300 -2.03984800 -1.88731200

O -5.15220100 -0.94935400 -3.42373900

Au -7.39747100 5.78775700 -2.25948100

S -0.81827500 -10.91242200 -4.98816700

C -1.59287100 -9.35536900 -4.67741600

C -2.74107900 -9.04398800 -5.42916100

N -1.09414400 -8.52308100 -3.75829800

C -3.37659000 -7.84035200 -5.20306200

H -3.11469800 -9.74402500 -6.16561800

C -1.72283700 -7.36223800 -3.55525900

C -2.86412500 -6.96655700 -4.24118700

H -4.26602500 -7.56474300 -5.75740300

H -1.29827000 -6.70641200 -2.80240600

C -3.52388300 -5.65672800 -3.94535000

O -4.56789800 -5.34921700 -4.58277600

O -3.02502900 -4.90480300 -3.06316200

Au 0.96090600 -10.90683200 -3.48706900

Ca -4.82567600 -3.32684000 -3.30065200

MNA-Ba

S -8.61818200 4.82982200 -0.93395800

C -7.87367300 3.25987100 -1.27562300

C -8.42581500 2.13220300 -0.64337300

N -6.83003600 3.17302800 -2.10390500

C -7.87134300 0.89245800 -0.89574000

H -9.27102600 2.24282600 0.02425800

C -6.30718400 1.96294400 -2.33010400

C -6.78069000 0.78788000 -1.76116500

H -8.26934500 -0.00277300 -0.43231500

H -5.45912100 1.92045600 -3.00572900

C -6.14298100 -0.54584300 -2.07589700

O -6.63880000 -1.56556700 -1.53629800

O -5.15979600 -0.55419800 -2.85757500

Au -7.39418300 6.28069500 -2.27767400

S -0.50273800 -11.27890600 -5.45139200

C -1.32388500 -9.78239900 -4.98010400

C -2.46150900 -9.41285800 -5.71829100

N -0.86706000 -9.05056900 -3.96112000

C -3.12706100 -8.25104800 -5.37670100

H -2.80154100 -10.03309800 -6.53800200

C -1.52909800 -7.93236800 -3.64600800

C -2.66180400 -7.47930100 -4.31044600

H -4.00679900 -7.93039100 -5.92277600

H -1.13946400 -7.35918500 -2.81139500

C -3.35381600 -6.20473300 -3.88426600

O -4.35461300 -5.83606300 -4.54715600

O -2.88651500 -5.59294900 -2.89006900

Au 1.27446600 -11.38661900 -3.95139800

Ba -4.85023400 -3.46784000 -2.86680400

MNA-Pb

S -6.92802900 1.75310300 0.79632800

C -6.64425400 0.80097400 -0.66626200

C -7.43550700 -0.28444800 -1.08035300

N -5.57935000 1.21515100 -1.36613900

C -7.10249000 -0.93994600 -2.24917300

H -8.28862200 -0.59688100 -0.49350700

C -5.27419700 0.57609800 -2.49193000

C -5.99798400 -0.50552700 -2.98374600

H -7.68701400 -1.78175900 -2.60127400

H -4.40757200 0.93481300 -3.03769100

C -5.59563800 -1.16130000 -4.25037600

O -6.23917000 -2.18701500 -4.64830900

O -4.63544300 -0.71717600 -4.92754300

Au -8.81892100 0.86125200 1.80630200

S -1.46977900 -8.95248200 -1.95949400

C -2.35487200 -7.77501700 -2.93665400

C -3.56347600 -8.03445800 -3.60588500

N -1.75622000 -6.57730100 -2.98307700

C -4.14780600 -7.01734700 -4.33376400

H -4.02429900 -9.01140000 -3.55047400

C -2.33231500 -5.60749000 -3.68839200

C -3.52722900 -5.76845900 -4.38306200

H -5.07941800 -7.17609000 -4.86409400

H -1.82355800 -4.64925500 -3.70619400

C -4.11580600 -4.64821100 -5.15377100

O -5.19372200 -4.79989800 -5.77974300

O -3.51411400 -3.52449500 -5.17786800

Au -2.67315000 -10.93553100 -2.03876400

Pb -5.01896400 -2.49285400 -6.55822900

MNA-CYS-Pb

S 2.14194300 -7.91521600 1.27829200

C 1.02486600 -6.68322200 2.04287100

C 1.25057000 -5.26062100 1.54426900

H -0.01172900 -6.97016700 1.88221800

H 1.24029200 -6.72900800 3.11396400

H 0.99613700 -5.24477000 0.46922600

N 2.61153100 -4.81585000 1.80784000

C 0.25819900 -4.29703400 2.17300400

H 3.25111700 -5.38642300 1.26308600

H 2.71953200 -3.85286400 1.50739300

O 0.59773400 -3.08064700 2.32446400

O -0.89565100 -4.67458700 2.48488700

Au 1.67139800 -7.68327300 -0.97808600

Pb -1.43396400 -2.38492200 3.13117500

S -1.83421000 0.96516600 -4.89302800

C -1.73502000 0.32705900 -3.25371200

C -2.33405900 -0.92391500 -3.00752200

N -1.10944500 1.02094100 -2.29587900

C -2.27402600 -1.45114500 -1.73543800

H -2.83148900 -1.45395100 -3.80951000

C -1.06080000 0.49677800 -1.07113500

C -1.62157700 -0.72879200 -0.73211900

H -2.72251700 -2.41029800 -1.50543300

H -0.54741500 1.07890300 -0.31276100

C -1.52345900 -1.24177700 0.65448000

O -2.03264700 -2.37859200 0.92794400

O -0.94738900 -0.57692900 1.55027000

Au -0.72054400 3.00366100 -4.70972400

MNA-CYS-Ba

S 2.35696600 -8.85243800 2.32940400

C 1.27699600 -7.39114200 2.57908600

C 1.93441300 -6.06113900 2.23316400

H 0.35838400 -7.51090400 2.01039900

H 1.03427900 -7.40646600 3.64584600

H 2.13602900 -6.07069300 1.14705600

N 3.14507600 -5.85875100 3.01950400

C 0.95939200 -4.88519600 2.43756900

H 3.88315700 -6.46181500 2.67180300

H 3.44193900 -4.89447300 2.91635900

O 1.43092300 -3.80066300 2.86130700

O -0.24328000 -5.06255100 2.12155100

Au 2.83126400 -8.74066100 0.06730300

S -2.92043100 2.09066600 -5.26583100

C -2.55558300 1.25149900 -3.75030900

C -3.25569400 0.06109100 -3.48755900

N -1.64908300 1.74676900 -2.90404800

C -2.99224700 -0.61855100 -2.31427400

H -3.98346800 -0.31008600 -4.19820300

C -1.41212600 1.07340000 -1.77298800

C -2.04654700 -0.11094200 -1.42130200

H -3.50779900 -1.54125900 -2.07495500

H -0.67194200 1.49833100 -1.10314800

C -1.72356800 -0.81462400 -0.12430000

O -2.31131100 -1.90015300 0.10858800

O -0.88894200 -0.27379800 0.64413600

Au -1.46086700 3.90245100 -5.21314400

Ba -1.03855200 -2.30939800 2.68802400

MNA-CYS-Ca

S 2.24032400 -8.38232800 2.07382900

C 1.10989700 -6.96506700 2.33813900

C 1.65578400 -5.62628900 1.85196600

H 0.14982500 -7.16314800 1.86789400

H 0.96718100 -6.92280000 3.42188400

H 1.73714600 -5.68443300 0.75185500

N 2.92494400 -5.31179000 2.49282100

C 0.64508400 -4.50781100 2.10820500

H 3.63981400 -5.94393200 2.14695400

H 3.19607600 -4.36444200 2.25214900

O 1.06621500 -3.36118400 2.41957800

O -0.58264200 -4.75498700 1.96387700

Au 2.55790000 -8.36358900 -0.21874800

S -2.45102800 1.67633200 -4.98543900

C -2.21107900 0.85352800 -3.44065100

C -2.97761500 -0.30091400 -3.19516700

N -1.32799000 1.32550300 -2.55500700

C -2.80486000 -0.97051700 -2.00121900

H -3.68438500 -0.65228900 -3.93621300

C -1.17493800 0.66304700 -1.40561900

C -1.87923400 -0.48735800 -1.07327000

H -3.37243100 -1.86509700 -1.77378900

H -0.45035300 1.06671800 -0.70602100

C -1.64771300 -1.18365500 0.23000900

O -2.31656200 -2.22117000 0.48866400

O -0.78877500 -0.72013500 1.02967600

Au -0.92791300 3.43361900 -4.86352100

Ca -1.16529500 -2.51730800 2.56820000

MNA-CYS-Cd

S 2.29274000 -8.69549900 2.61636200

C 1.18258300 -7.23810300 2.58795000

C 1.87669600 -5.94271800 2.18496400

H 0.34011400 -7.42764700 1.92746700

H 0.81145400 -7.14690600 3.61273600

H 2.20852200 -6.06378000 1.13835100

N 2.97834000 -5.63384000 3.08708000

C 0.89371200 -4.77324300 2.13823800

H 3.71431900 -6.32126300 2.95831700

H 3.35455100 -4.72113500 2.85412200

O 1.31532200 -3.61690100 2.39924700

O -0.29518200 -4.98940100 1.78436300

Au 3.07137600 -8.77815500 0.43735200

S -3.12519200 2.35389900 -4.87052300

C -2.62786100 1.32391700 -3.52453200

C -3.38972500 0.16704600 -3.27695200

N -1.56347100 1.64911900 -2.78408600

C -3.02482400 -0.65672700 -2.23230400

H -4.24691800 -0.06316700 -3.89678200

C -1.22682800 0.84026500 -1.77643300

C -1.91536200 -0.32220100 -1.45208500

H -3.58694400 -1.55488100 -2.00590000

H -0.36096300 1.13043900 -1.19035300

C -1.49496100 -1.17125000 -0.29669200

O -2.15426400 -2.21512700 -0.04437100

O -0.50444300 -0.81713900 0.39678900

Au -1.60412800 4.11339400 -4.74826800

Cd -0.79128700 -2.68049200 1.82698000

MNA-CYS-Mg

S 2.38922800 -8.24559900 3.23968700

C 1.28131400 -6.80356100 3.03645600

C 1.69534800 -5.86404200 1.90414000

H 0.25790500 -7.13731200 2.88118400

H 1.33892000 -6.26596000 3.98720300

H 1.59439500 -6.42813300 0.96122700

N 3.03532100 -5.34516300 2.12238100

C 0.69229900 -4.73074200 1.78322400

H 3.69765100 -6.11180700 2.05489500

H 3.26731500 -4.66836000 1.40338800

O 1.07057600 -3.53107400 1.69304800

O -0.54311200 -5.00555100 1.73473300

Au 2.23748800 -9.28499500 1.17481200

S -2.74886500 2.44740600 -4.61752200

C -2.39314600 1.31952700 -3.31081000

C -2.86980800 0.00101700 -3.44785300

N -1.69576400 1.72033000 -2.24160700

C -2.60093300 -0.90952300 -2.44881700

H -3.43273600 -0.28358900 -4.32743400

C -1.45116700 0.82849600 -1.28094900

C -1.87332400 -0.49464900 -1.32967100

H -2.94408400 -1.93492700 -2.51786800

H -0.88349400 1.17902600 -0.42500900

C -1.54649100 -1.43492000 -0.23205300

O -1.86792200 -2.65596200 -0.33131900

O -0.93723000 -1.02911200 0.80174200

Au -1.70328200 4.40418500 -3.91125000

Mg -0.93902600 -2.98650500 1.49507800
